# Efficacy of a Digital Mental Health Biopsychosocial Transdiagnostic Intervention With or Without Therapist Assistance for Adults With Anxiety and Depression: Adaptive Randomized Controlled Trial

**DOI:** 10.2196/45135

**Published:** 2023-06-12

**Authors:** Brooke Andrews, Britt Klein, Huy Van Nguyen, Denise Corboy, Suzanne McLaren, Shaun Watson

**Affiliations:** 1 Health Innovation & Transformation Centre Federation University Australia Ballarat Australia; 2 Biopsychosocial & eHealth Research & Innovation Federation University Australia Ballarat Australia; 3 Blue Sky Mind Research Consultancy Ballarat Australia; 4 Faculty of Business, Justice and Behavioural Sciences School of Psychology Charles Sturt University Port Macquarie Australia

**Keywords:** video chat therapy, therapist assistance, self-help, transdiagnostic, digital intervention, anxiety, depression, comorbidity

## Abstract

**Background:**

Digital mental health (DMH) interventions incorporating elements that adapt to the evolving needs of consumers have the potential to further our understanding of the optimal intensity of therapist assistance and inform stepped-care models.

**Objective:**

The primary objective was to compare the efficacy of a transdiagnostic biopsychosocial DMH program, with or without therapist assistance for adults with subthreshold symptoms or a diagnosis of anxiety or depression.

**Methods:**

In a randomized adaptive clinical trial design, all participants had access to the DMH program, with eligibility to have their program augmented with therapist assistance determined by program engagement or symptom severity. Participants who met stepped-care criteria were randomized to have their treatment program augmented with either low-intensity (10 min/week of video chat support for 7 weeks) or high-intensity (50 min/week of video chat support for 7 weeks) therapist assistance. A total of 103 participants (mean age 34.17, SD 10.50 years) were assessed before (week 0), during (weeks 3 and 6), and after the intervention (week 9) and at the 3-month follow-up (week 21). The effects of 3 treatment conditions (DMH program only, DMH program+low-intensity therapist assistance, and DMH program+high-intensity therapist assistance) on changes in the 2 primary outcomes of anxiety (7-item Generalized Anxiety Disorder Scale [GAD-7]) and depression (9-item Patient Health Questionnaire [PHQ-9]) were assessed using the Cohen *d*, reliable change index, and mixed-effects linear regression analyses.

**Results:**

There were no substantial differences in the outcome measures among intervention conditions. However, there were significant time effect changes in most outcomes over time. All 3 intervention conditions demonstrated strong and significant treatment effect changes in GAD-7 and PHQ-9 scores, with absolute Cohen *d* values ranging from 0.82 to 1.79 (all *P*<.05). The mixed-effects models revealed that, in the Life Flex program–only condition at week 3, mean GAD-7 and PHQ-9 scores significantly decreased from baseline by 3.54 and 4.38 (all *P*<.001), respectively. At weeks 6, 9, and 21, GAD-7 and PHQ-9 scores significantly decreased from baseline by at least 6 and 7 points (all *P*<.001), respectively. Nonresponders at week 3 who were stepped up to therapist assistance increased program engagement and treatment response. At the postintervention time point and 3-month follow-up, 67% (44/65) and 69% (34/49) of the participants, respectively, no longer met diagnostic criteria for anxiety or depression.

**Conclusions:**

The findings highlight that early detection of low engagement and non–treatment response presents an opportunity to effectively intervene by incorporating an adaptive design. Although the study findings indicate that therapist assistance was no more effective than the DMH intervention program alone for reducing symptoms of anxiety or depression, the data highlight the potential influence of participant selection bias and participant preferences within stepped-care treatment models.

**Trial Registration:**

Australian New Zealand Clinical Trials Registry ACTRN12620000422921; https://www.anzctr.org.au/Trial/Registration/TrialReview.aspx?id=378317&isReview=true

**International Registered Report Identifier (IRRID):**

RR2-10.2196/45040

## Introduction

### Background

Digital health interventions have a well-established evidence base for the effective delivery of mental health care for various psychological disorders, including anxiety and depression [1-4]. Digital mental health (DMH) interventions adapt standard treatment protocols for delivery over the internet, presenting an opportunity to scale and disseminate psychological treatment more widely [5,6]. The most studied form of therapeutic orientation within DMH intervention programs for anxiety and depression is cognitive behavioral therapy (CBT), whereby psychoeducation, cognitive and behavioral skills, and relapse prevention content are delivered via the internet through modules and practice exercises, commonly referred to as internet-delivered CBT [1,3,7-10]. DMH intervention research has extended to studying transdiagnostic CBT treatment protocols because of the widely acknowledged comorbidity of anxiety and depression, with results showing large symptom improvement similar to face-to-face treatment and disorder-specific treatment programs [4,9,11-14].

DMH intervention programs have been disseminated in a variety of formats, from purely self-help (ie, no therapist assistance or other form of human involvement) to guided (ie, therapist assistance) delivery methods in which the mode and amount of therapist involvement can vary substantially among studies [15,16]. The addition of a therapist to a web-based DMH intervention program typically provides participants with support designed to enhance motivation and engagement with the program content. Previous research, including systematic reviews and meta-analyses for anxiety and depression, have identified that DMH intervention programs with therapist assistance lead to lower attrition, higher engagement, and increased clinical outcomes with moderate to large effect sizes compared with studies without therapist assistance [1,2,7,11,17-19]. However, other studies have found no substantial difference in outcomes when a therapist was added to a DMH intervention program [9,20-22]. When research findings are examined more closely, it appears that the predictors of engagement vary across studies irrespective of the presence and frequency of the therapist assistance provided [23]. On the basis of the findings of a systematic review conducted on the evaluation of adaptive elements within internet-delivered psychological treatment, researchers [23] proposed that the ability of a DMH intervention to adapt to the participant substantially affects engagement and treatment outcomes and, therefore, warrants further investigation.

Research on DMH intervention programs incorporating adaptive treatment elements has the potential to further our understanding of the optimal intensity of therapist assistance. Adaptive intervention designs incorporate criteria that influence how and whether treatment is maintained or augmented based on participant responses, such as engagement levels or symptom severity [24,25]. An adaptive intervention design that has been studied is stepped-care models. Stepped-care models enable participants with evolving needs to receive higher-intensity treatment if required [5,24,25]. Previous research, including systematic reviews and meta-analyses conducted on stepped care, indicates that stepped care is an effective delivery model with a moderate effect size for improving depressive symptoms and disorders [26,27] and a considerably better model than care as usual for reducing anxiety symptoms, as well as having significantly higher response rates for anxiety disorders [28]. Within a stepped-care model, a DMH intervention program is typically offered as part of the first step of *self-management* (ie, self-help web-based program). If or when required (ie, a person’s needs change or symptoms rise), the intensity of treatment can be increased, for example, by adding a therapist to assist while using a web-based DMH intervention program. Adaptive intervention research designs provide the opportunity to evaluate the first 2 stages of a stepped-care model as the type or dosage of treatment can be adapted according to decision criteria [24,25].

Therapist assistance within DMH intervention programs has been delivered synchronously and asynchronously. Synchronous interactions include telephone calls, instant messaging, and video chat interactions between a therapist and participant in real time; asynchronous communication can also include messaging and email support [2]. To date, the therapist assistance literature has largely focused on evaluating the effectiveness of telephone and email modalities [21,29-31], with research into the effectiveness of support delivered via video chat technology being an opportunity for further investigation. To date, the video chat literature has predominately focused on establishing the effectiveness of the video chat modality through comparison with face-to-face treatment, with results being comparable and showing high levels of satisfaction and acceptability [2,32-35].

There is currently a lack of research on the use of video chat as a modality of therapist assistance within a DMH intervention. To our knowledge, there is no identified adaptive randomized clinical trial that has investigated the efficacy of a 2-stage adaptive design whereby a transdiagnostic biopsychosocial DMH intervention program is augmented with either low- or high-intensity video chat–based therapist assistance should a participant not engage with the program or their symptoms not improve. In stepped-care research, little is known about the intensity of therapist assistance required at the next level of care. To further understand the efficacy of stepped-care models and adaptive intervention designs within DMH interventions, this clinical trial sought to evaluate and compare no therapist assistance with participants randomized to receive either 10 minutes (low intensity) or 50 minutes (high intensity) of therapist assistance per week. In this study, both low- and high-intensity therapist assistance were delivered via video chat technology. This further contributes to the body of literature as higher-intensity therapist assistance is typically delivered in person within stepped-care models.

### Objectives

The primary aim of this study was to investigate and comparatively evaluate the efficacy of various support intensities of the Life Flex DMH intervention program for adults with subthreshold symptoms or a diagnosis of anxiety or depression. Specifically, the study sought to evaluate whether augmenting a self-help DMH intervention program with low- or high-intensity therapist assistance delivered via video chat technology improved the clinical outcomes of anxiety and depression compared with a DMH intervention program only. Participant secondary outcomes of quality of life, social support, sleep, and physical and mental health ratings were reported, as well as program engagement (the number of log-ins, percentage of program completed, and number of pages viewed), acceptability (satisfaction survey), and usability of the Life Flex intervention program. Predictor outcomes of motivation and self-efficacy will be evaluated in another study.

## Methods

### Participants and Recruitment

Recruitment took place between November 2020 and March 2022 via social media advertisements, health websites, and information flyers sent to medical clinics. All applicants were directed to the Life Flex study website created for the trial, in which they completed a 2-step screening process. All applicants gave their consent to take part in the study by reading a Plain Language Information Statement on the web and clicking a checkbox that said the following: “I have read the Plain Language Information Statement and I agree to the above conditions.” The Plain Language Information Statement informed participants of how long each diagnostic assessment and questionnaire would take, along with the estimated weekly time required to complete the intervention. Participants were also provided with detailed information regarding data protection and storage procedures. The aims of the study were not stated, no formal participant debriefing was undertaken, and the clinical trial adhered to the CONSORT-EHEALTH (Consolidated Standards of Reporting Trials of Electronic and Mobile Health Applications and Online Telehealth) checklist [36] (Multimedia Appendix 1). Upon registering their interest in the trial, applicants completed a digitally based screening form that inquired about their age, location, and current mental health service involvement, followed by a semistructured telephone interview to confirm eligibility and the presence of anxiety or depression symptoms. For eligible participants, a video chat assessment was scheduled as a screening and diagnostic tool for further evaluation of anxiety or depression symptoms.

Participants were eligible for the trial if they met the following criteria: aged ≥18 years, Australian residents, access to the internet, ability to register their interest on the web using an email address, ability to read and write in English, and symptoms or diagnosis of anxiety or depression (as assessed using the Mini International Neuropsychiatric Interview 7.0.2) [37]. Participants with subthreshold symptoms were included, as is typical in stepped-care models, to provide an opportunity for earlier intervention. Participants were excluded if they were receiving psychological treatment for their mental health, had moderate to severe levels of alcohol or substance use, had active psychosis or active suicidal intent or plans, had unstable bipolar disorder, or had unstable doses of medication.

### Design

A pre-during-post–follow-up randomized adaptive trial design was used to explore the various support intensities of the Life Flex program. Participants were assessed via a closed survey accessible via their private log-in on the DMH website before (week 0), during (weeks 3 and 6), and after the intervention (week 9) and at the 3-month follow-up (week 21). The web-based questionnaire was created using electronic forms on the DMH website. The web-based questionnaire and associated participant flow were tested for functionality via the creation of dummy participants before the trial commenced. Participants were required to complete the preintervention and week 3 during-the-intervention questionnaires to gain access to the Life Flex program. Participants were not required but were encouraged to complete the week 6 during-the-intervention questionnaire; however, they were incentivized with an Aus $10 (US $6.77) gift card voucher for completion of the postintervention (week 9) and 3-month follow-up (week 21) questionnaires. Duplicate entries from the same participant were not possible as the questionnaire was not displayed a second time. If participants endorsed suicidal ideation on the 9-item Patient Health Questionnaire (PHQ-9) [38], they received a pop-up notification on the screen directing them to seek further assistance. Participants were also sent an automated email encouraging them to seek more support via their general practitioner or one of the emergency contact phone lines (all details were provided in the email). On their dashboard, participants also had access to emergency contact support numbers and a safety plan that they could complete. If any risk was identified during the postintervention or 3-month follow-up diagnostic assessment, therapists completed a risk assessment and safety plan where required. All data were deidentified before analysis.

### Ethics Approval

This study received ethics approval from the Federation University human research ethics committee (approval A19-095) and was preregistered with the Australian New Zealand Clinical Trials Registry (ANZCTR; ACTRN12620000422921).

### Outcome Intervention Conditions

#### DMH Intervention Program Only

Treatment was delivered within a secure and encrypted digitally based treatment platform and comprised the primary intervention for all 3 treatment conditions: a third-wave, transdiagnostic biopsychosocial DMH intervention program called Life Flex. The treatment in step 1 of the trial comprised the Life Flex program only, whereby participants completed the self-help program Life Flex without therapist assistance. All participants received automated emails to inform them of the module content as well as the release and reminders of scheduled assessments. Participants completed the program on a scheduled release design, with a new module being released every week except for module 4 (participants were given 2 weeks to work through and practice module 4 content). Participants who improved or engaged with the Life Flex program within the first 3 weeks maintained their no-therapist-assistance DMH intervention program.

The aim of the Life Flex program is to increase psychological flexibility and positive affect in anxiety and depression. The program incorporates CBT, emotion regulation strategies, and neuroplasticity principles. The Life Flex program is suitable for individuals with or without a diagnosis of anxiety or depression and contains 6 core modules along with an introductory and booster module. Each module of the program takes approximately 25 minutes to complete. In addition, to reinforce the module-based information, there are 20 to 30 minutes of offline activities each week.

Offline activities include applying the concepts and techniques discussed in the modules (eg, self-monitoring of depressive and anxiety symptoms), undertaking one of the biological and wellness flexibility intervention strategies, monitoring emotions and thoughts, and undertaking the gradual exposure or behavioral activation activities. Participants also receive automated emails (eg, to remind them to log on or complete the questionnaires) and are asked several questions at the beginning of each module to help gauge their progress. Module features include text, graphics, audio, video, editable forms, web-based games (eg, cognitive bias modification), and downloads. Modules are accessible via the web on mobile or tablet devices. Further information regarding the modules of the Life Flex program is presented in Multimedia Appendix 2.

#### DMH Intervention Program and Low-Intensity Therapist Assistance

Participants who were stepped up to the low-intensity therapist-assisted treatment condition completed the same Life Flex program augmented with 10 minutes per week of video chat support for 7 weeks with a therapist for support and progress review of program content. Therapists were encouraged to display warmth and unconditional positive regard and were required to manage any risks (eg, suicidal thoughts) that arose.

#### DMH Intervention Program and High-Intensity Therapist Assistance

Participants who were stepped up to the high-intensity therapist-assisted treatment condition also completed the same Life Flex program augmented with 50 minutes per week of video chat support for 7 weeks with a therapist for individualized tailoring of the program, questionnaire feedback, and discussion of module content (ie, examination of unhelpful cognitions and support in completing exposure tasks or behavioral activation), as well as skill generalizability beyond the Life Flex program. Similar to the low-intensity condition, therapists were encouraged to be supportive and manage any issues of risk.

The therapist assistance in both treatment conditions was manualized via case note templates, which included session agendas used by the therapists. In all 3 treatment conditions, participants had access to the Life Flex program for a further 14 weeks after the last module release.

### Assessors and Therapists

The assessors and therapists (n=22) were registered provisional and generalist psychologists who were undertaking a postgraduate clinical placement within a community mental health service. Each assessor and therapist had undertaken coursework in clinical psychology, including psychological assessment and interventions, as well as completing a purposively developed 14-hour, 5-module web-based training program covering DMH interventions (specifically video chat technology), diagnostic assessments, qualitative interviewing techniques, and transdiagnostic CBT principles that underpin the Life Flex program. The training program comprised a 40-item multiple-choice competency assessment on which therapists were required to obtain a minimum score of 80%.

All assessors were required to demonstrate competency in diagnostic assessments before administration. Both assessors and therapists received daily training and supervision throughout the trial from the first author, an experienced clinical psychologist. Assessment and therapist sessions were audio or video recorded (with participant permission) so that random checking (20% of the total) could be completed to ensure fidelity to administration, enable agreement and interrater reliability of diagnoses, and ensure that fidelity to the therapist’s role (low or high intensity) was maintained. At least one treatment session was reviewed for each therapist. An adapted version of the Internet-Delivered Cognitive Behavior Therapy–Therapist Rating Scale [39] and the original version of the Cognitive Therapy Rating Scale [40] was used by supervisors in the review process to ensure treatment fidelity in both therapist-assisted conditions. A new assessor was assigned for all 3 assessment time points and differed from the therapist assigned to participants in either the low- or high-intensity therapist-assisted conditions.

### Procedure

Diagnostic assessment interviews were conducted before the intervention (week 0), after the intervention (week 9), and at the 3-month follow-up (week 21), with no more than 3 attempts made to contact participants. Outcomes were assessed before the intervention (week 0), during the intervention (weeks 3 and 6), after the intervention (week 9), and at the 3-month follow-up (week 21). The web-based questionnaire assessment comprised a demographic questionnaire, the 7-item Generalized Anxiety Disorder Scale (GAD-7) [41], the PHQ-9 [38], the Client Motivation for Therapy Scale (CMOTS) [42], a modified version of the Bipolar Self-Efficacy Scale [43], the Assessment of Quality of Life (AQol-4D) [44], the Working Alliance Inventory–Short (WAI-S) [45], the System Usability Scale (SUS) [46], and a purposively developed 10-item satisfaction questionnaire. Before the intervention, participants were also asked to rate their treatment preference in order (should they have been given a choice) for DMH intervention program only, low-intensity therapist assistance, or high-intensity therapist assistance.

### Stepped-Care Rules

All eligible participants were given access to the Life Flex program. Symptoms and program engagement were evaluated at week 3 to assess whether the stepped-care rules were met for participants to have their treatment program augmented with therapist assistance. Stepped-care rules were met if participants’ symptoms deteriorated by >5 points (as assessed through comparison of their baseline depression [PHQ-9] [38] and anxiety [GAD-7] [41] scores against their week 3 scores), participants showed symptom improvement but scores remained in a severe range, participants did not complete the preintervention or week 3 questionnaire, or they did not engage with the Life Flex program (ie, noncompletion of the introduction or module 1).

### Attrition

In terms of attrition, 8.7% (9/103) of the participants failed to complete the introduction module and module 1 of the program by the 3-month follow-up. In terms of scheduled assessment completions, 8.7% (9/103) of the participants failed to complete at least one scheduled assessment following the preintervention assessment. Of the 103 participants who commenced the intervention, 38 (36.9%) did not attend the postassessment interview, and 54 (52.4%) did not attend the 3-month follow-up assessment interview.

### Randomization

A block randomization design was used to randomly allocate participants who met the stepped-care rules to either the low- or high-intensity therapist-assisted condition. Both the study participants and therapists were aware of what therapist-assisted condition they had been allocated to; however, they remained unaware of the study’s aims.

### Participant Flow

**Figure 1:**
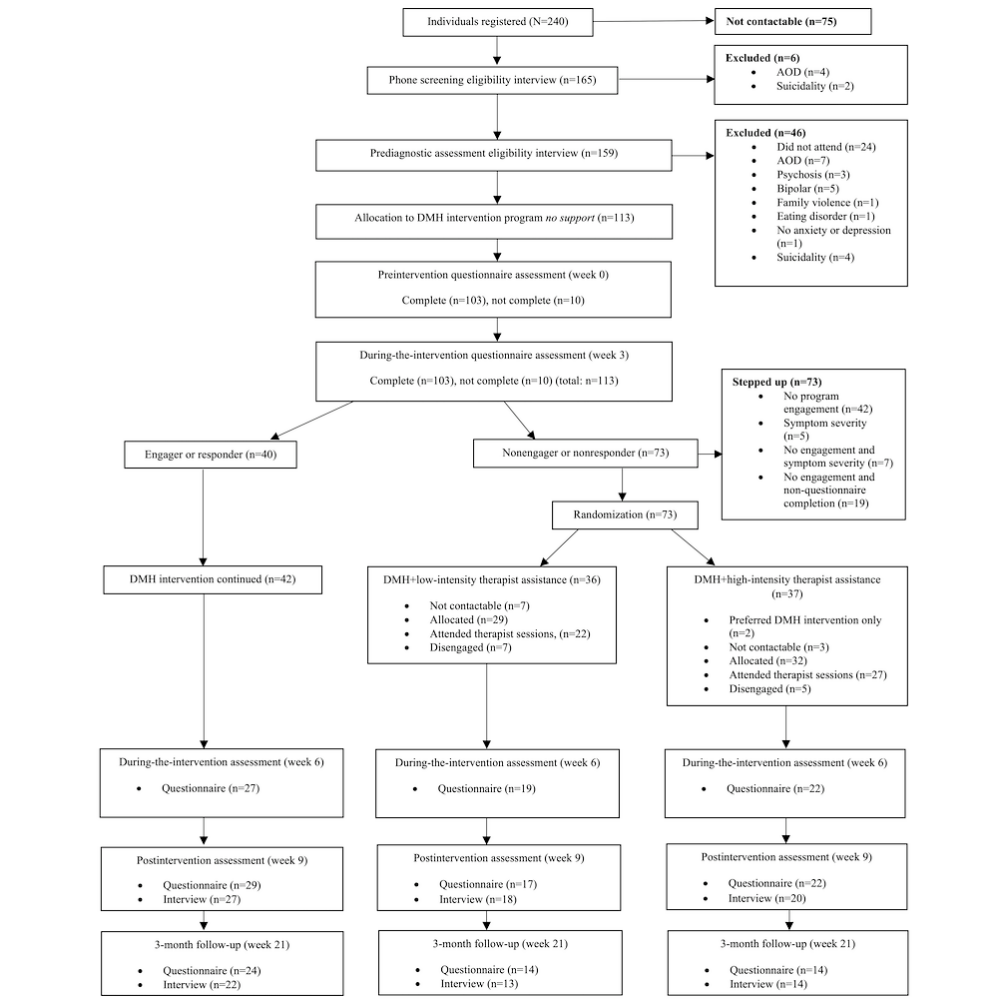
Participant flow chart. AOD: alcohol and other drugs; DMH: digital mental health.

A total of 240 individuals registered to participate in the adaptive clinical trial during the recruitment period. Of the 240 applicants, 75 (31.3%) were not contactable after registration, 6 (2.5%) were excluded during the prescreening phone interview stage, and a further 46 (19.2%) were excluded during the prediagnostic assessment interview because they met the exclusion criteria (see the exclusions in Figure 1), leaving 113 (47.1%) applicants who met all inclusion criteria. All 113 participants were given access to the preintervention assessment questionnaire before commencing the Life Flex program. Before the intervention and at week 3, a total of 8.8% (10/113) of the participants had not completed the preintervention questionnaire and, thus, were considered eligible for randomization to therapist assistance, along with a further 55.8% (63/113) of the participants (see the stepped-care criteria met by the participants in Figure 1).

Of the 73 participants who met the stepped-care criteria, 36 (49%) were randomized to low-intensity therapist assistance, and 37 (51%) were randomized to high-intensity therapist assistance. Of the 36 participants allocated to low-intensity therapist assistance, 7 (19%) remained uncontactable, and 7 (19%) scheduled therapist-assisted sessions but did not attend, leaving 22 (61%) who actively attended low-intensity therapist-assisted sessions adjunctive to the Life Flex program. Of the 37 participants allocated to high-intensity therapist assistance, 3 (8%) remained uncontactable, 2 (5%) preferred to stay in the DMH intervention program–only condition, and 5 (14%) scheduled therapist-assisted sessions but did not attend, leaving 27 (73%) who actively attended high-intensity therapist-assisted sessions adjunctive to the Life Flex program.

Progress on the Life Flex program of the 16% (12/73) of participants who did not attend the scheduled therapist sessions was also assessed. Of these 12 participants, 1 (8%) engaged with the program for a further 3 days after the offer of therapist assistance, and 1 (8%) engaged for a further week before disengaging from the Life Flex program. A total of 35.4% (40/113) of the participants did not meet the stepped-care criteria at week 3; however, 37.2% (42/113) of the participants continued in the DMH intervention program–only condition as 1.8% (2/113) of the participants, who were initially randomized to high-intensity therapist assistance, preferred to remain in the program-only condition. Figure 1 illustrates the participants’ pathways through the clinical trial.

### Measures

#### Primary Outcome Measures

The primary self-report outcomes were anxiety severity according to the GAD-7 [41] and depression severity according to the PHQ-9 [38]. The GAD-7 [30] total scores range from 0 to 21, with higher scores indicating more severe anxiety symptoms using a clinical cutoff of 8. The PHQ-9 [38] total scores range from 0 to 27, with higher scores indicating greater depressive symptoms using a clinical cutoff of 10. Both the GAD-7 and PHQ-9 have demonstrated sensitivity in detecting treatment change in transdiagnostic internet interventions [11,47,48].

#### Secondary Outcome Measures

##### Motivation

The CMOTS [42] is a 24-item self-report measure designed to assess the degree to which an individual is motivated for therapy and the impact of a person’s motivation on treatment effectiveness and mental health symptoms. Items assess 6 dimensions of the self-determination continuum of motivation proposed by Deci and Ryan [49]: intrinsic motivation, 4 forms of extrinsic motivation (integrated, identified, introjected, and external regulation), and amotivation for therapy. The CMOTS obtains subscale scores for each type of motivation, with higher scores reflecting higher levels of each type of motivation.

##### Self-efficacy

The wording of the Bipolar Self-Efficacy Scale [43] was slightly modified for use in this study to measure the self-efficacy of participants with anxiety or depressive symptoms as opposed to bipolar disorder symptoms. The 17 items assess how confident an individual feels in performing a range of activities related to their mental health (ie, taking their medication as prescribed).

##### Working Alliance

The WAI-S [45] is an abbreviated version of the Working Alliance Inventory based on the theory by Bordin [50] and comprises the following subscales: goal agreement, task agreement, and bond. In the clinical trial, the WAI-S was used in a therapist version (10-item scale) and a participant version (12-item scale), with both versions used to capture the strength of the therapeutic alliance from both perspectives. Total scores range from 12 to 60, with higher scores indicating a stronger therapeutic alliance. To assess participants’ technological alliance with the self-help Life Flex program, the wording of the items on the WAI-S was slightly modified for this study.

##### Health Status

The AQol-4D [44] is a 12-item self-report measure designed to assess ratings of health-related quality of life in a variety of life areas, such as independent living, illness, social relationships, psychological well-being, and physical senses. The AQol-4D has been validated in several countries, has been shown to have a high degree of internal consistency (Cronbach α=.81), and is deemed an acceptable measure of health-related quality of life.

##### Program Usability

The SUS [46] is a 10-item measure designed to assess participant perceptions of the usability of a program. The SUS was used in this study to assess participants’ ease and perceptions of the Life Flex program’s usability (eg, “I thought the program was easy to use”). Each item is rated on a 5-point scale, with higher scores reflecting increased system usability. Scores between 0 and 50 are assessed as not acceptable, scores from 50 to 70 are considered marginal, and scores between 70 and 100 are considered acceptable. In previous studies, the SUS has been found to have an average score of 68 [35].

##### Treatment Satisfaction

A purposively designed 10-item satisfaction questionnaire was developed to assess participants’ level of satisfaction with the treatment received. In total, 8 of the 10 items are rated on a 4-point Likert scale. For the other 2 questions, participants are asked to qualitatively share what they believe are the most preferable parts of the Life Flex program, as well as what they believe are the least preferable parts of the Life Flex program.

##### Health Care Use

Participants completed a health care resource use questionnaire designed for the trial to evaluate primary and secondary health care consultations and medication use. All assessors and trial therapists also completed a resource questionnaire logging the time spent interacting with participants during the trial.

### Treatment Preferences

Before participation at baseline, the participants’ treatment preference was assessed by asking which of the 3 treatment options being evaluated in the trial they would prefer if they were given a choice. Participants ranked the 3 options of Life Flex program only, Life Flex program+low-intensity therapist assistance, and Life Flex program+high-intensity therapist assistance from most preferred to least preferred. Of the 103 participants, 38 (36.9%) received their first treatment preference, 34 (33%) received their second treatment preference, and 31 (30.1%) received their third treatment preference. Overwhelmingly, across the total sample (n=103), the first treatment preference was for high-intensity therapist assistance (n=61, 59.2%) followed by low-intensity therapist assistance as the second preferred treatment (n=68, 66%), with the Life Flex program only rated as the least preferred treatment (n=70, 68%). The preference data were further examined according to participants who did and did not meet the stepped-care criteria. Of the 63 participants who met the stepped-care criteria, 43 (68%) preferred high-intensity therapist assistance, 11 (17%) preferred low-intensity therapist assistance, and 9 (14%) preferred the DMH program only. Of the participants who remained in the program-only condition, 45% (18/40) preferred high-intensity therapist assistance, 35% (14/40) preferred low-intensity therapist assistance, and 20% (8/40) preferred the DMH program only. The remaining treatment preferences are presented in Multimedia Appendix 3.

### Diagnostic Status

All participants were administered video-based diagnostic interviews using the Mini International Neuropsychiatric Interview 7.0.2 [37] at the preintervention time point to determine whether they met the Diagnostic and Statistical Manual of Mental Disorders [51] criteria for an anxiety or depressive disorder and then again at the postintervention time point and 3-month follow-up. The clinical severity rating scale of 0 to 8 was applied to the diagnoses, with a clinical cutoff of 4 used to determine the diagnostic threshold. These assessments were conducted by the provisional and generally registered psychologists and averaged 64 (SD 25.05) minutes per participant for the preintervention assessment, 51 (SD 19.12) minutes for the postintervention assessment, and 24 (SD 29.30) minutes for the 3-month follow-up assessment.

### Statistical Analysis

#### Analysis of Intervention Effects

Stata (version 17; StataCorp) [52] and SPSS (version 26; IBM Corp) [53] were used to analyze the data. Categorical and continuous data were presented in numeric and percentage forms and mean and SDs where appropriate, respectively. ANOVA was used to compare the mean difference among the 3 groups (Multimedia Appendix 4). To compare categorical variables related to sociodemographic characteristics across groups (DMH intervention program only and low- and high-intensity therapist assistance), chi-square test statistics were computed (Multimedia Appendix 3).

**Figure 2:**
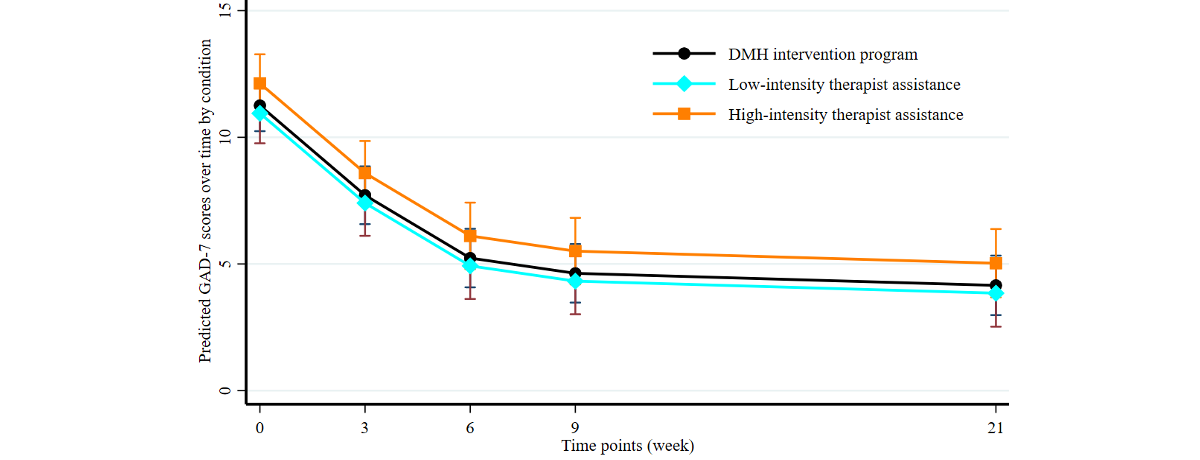
Trajectories of estimated mean 7-item Generalized Anxiety Disorder Scale (GAD-7) scores over time points by intervention conditions. DMH: digital mental health.

**Figure 3:**
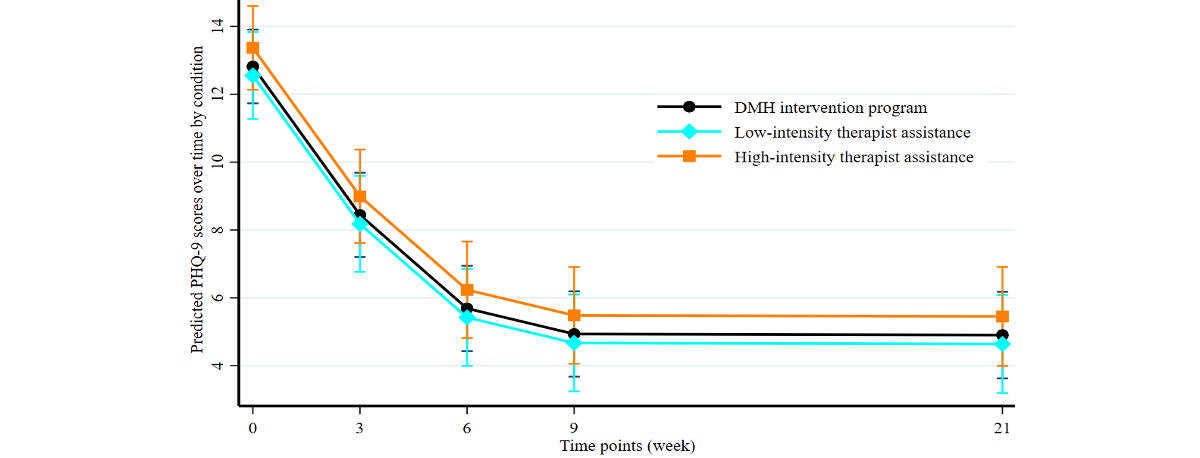
Trajectories of estimated mean 9-item Patient Health Questionnaire (PHQ-9) scores over time points by intervention conditions. DMH: digital mental health.

Treatment effects from preintervention to during-the-intervention, postintervention, and follow-up assessments were evaluated using a series of 1-tailed paired-sample *t* tests or Wilcoxon signed rank tests where applicable (Multimedia Appendix 5). All the intervention outcome measures were normally distributed except for mental health rating at the preintervention time point, the GAD-7 and PHQ-9 scores at weeks 9 and 21, the utility index, sleeping time, and physical health rating (week 21), so nonparametric test statistics such as Wilcoxon signed rank tests were applied where appropriate. The effect of intervention time points overall and by condition was assessed using ANOVA for effect size (Multimedia Appendix 5). The Cohen *d* classification scheme (small effect=0.2, medium effect=0.5, and large effect=0.8) was applied to index and interpret the size of the standardized difference (Multimedia Appendix 5). The effect size of intervention time points (preintervention, postintervention, and follow-up time points) was estimated using repeated-measure ANOVA (Multimedia Appendix 5). The reliable change index (a threshold) was calculated based on the Cronbach α at different time points and on baseline SD. Therefore, 5.06 for pre- and post- and 4.53 for pre- and follow-up treatment, respectively, was estimated for anxiety (GAD-7 Cronbach α at the preintervention, postintervention, and follow-up time points was .87, .88, and .93, respectively, and the baseline SD was 5.16), and 6.02 for pre- and post- and 4.55 for pre- and follow-up treatment, respectively, was estimated for depression (PHQ-9 Cronbach α at the preintervention, postintervention, and follow-up time points was .81, .84, and .99, respectively, and the baseline SD was 5.19). Clinically significant change based on literature was identified as 8.0 and 10.0 for the GAD-7 [54] and PHQ-9 [38], respectively. As a result, those participants who satisfied the threshold for both reliable change and clinically significant change were considered as a reliable and clinically significant change. We illustrated these data in Figures 2 and 3 and Multimedia Appendices 6-11.

**Figure 4:**
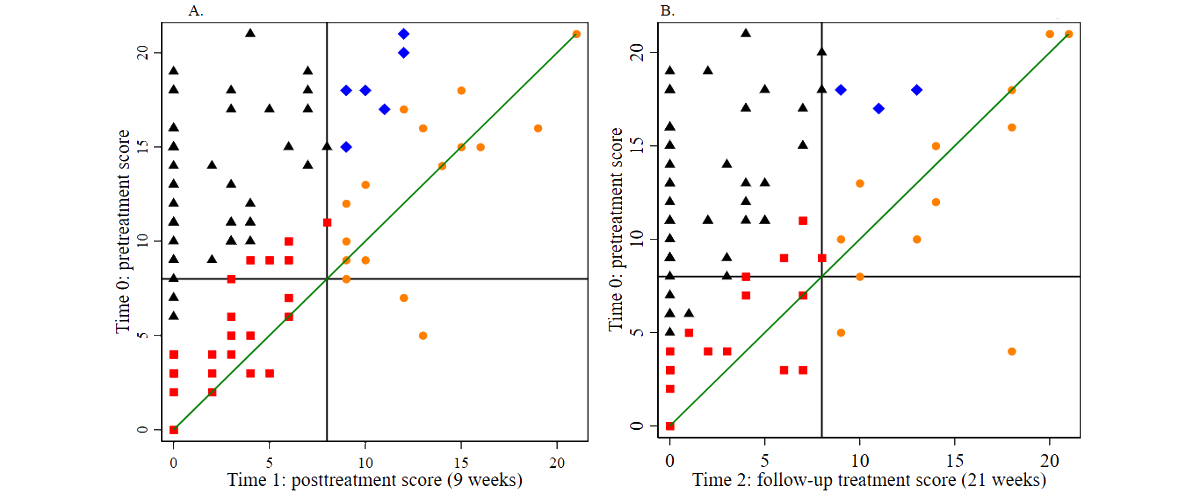
Reliable and clinically significant change in 7-item Generalized Anxiety Disorder Scale (GAD-7) score. (A) Change in GAD-7 scores from before to after treatment; (B) Change in GAD-7 scores from before treatment to follow-up.

**Figure 5:**
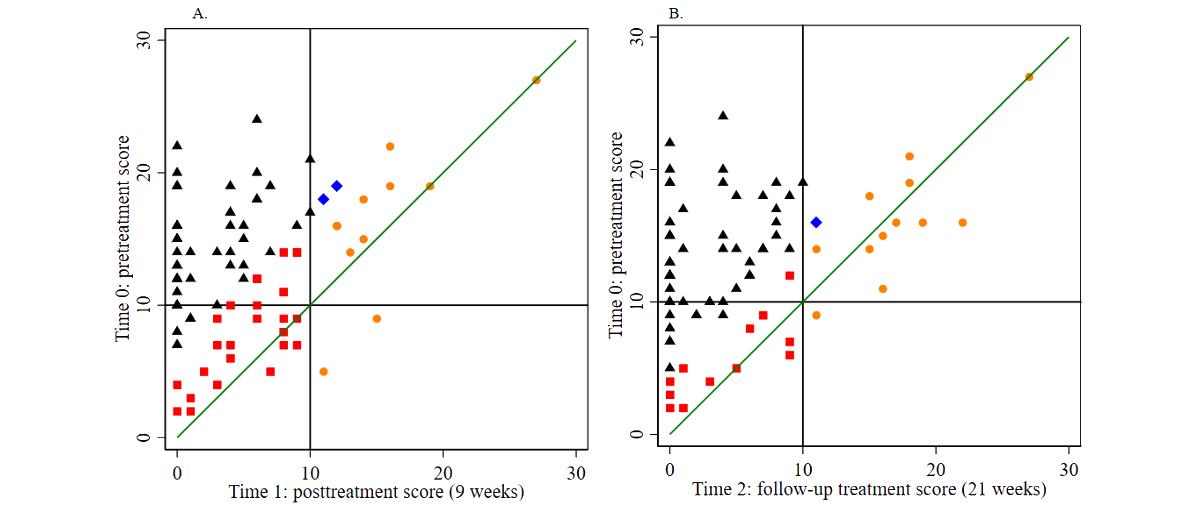
Reliable and clinically significant change in 9-item Patient Health Questionnaire (PHQ-9) score. (A) Change in PHQ-9 scores from before to after treatment; (B) Change in PHQ-9 scores from before treatment to follow-up.

Adjusted analysis of intervention effects was performed using mixed-effects linear regression models. We estimated both fixed and random effects to assess the intervention effects of time point and condition on the change in the 2 primary outcomes, GAD-7 and PHQ-9 scores. Both measures, GAD-7 and PHQ-9 in long format, were normally distributed, satisfying the assumption of the mixed-effects model. We also tested the interaction effects of time point and condition, but they were not significant; therefore, noninteractive models were assessed for both the GAD-7 and PHQ-9. The appropriateness of the mixed-effects model for each outcome was assessed by comparing each with the standard linear regression model and random-intercept linear model based on likelihood ratio tests with strong evidence (*P*<.05). We illustrated the changes in those outcomes in Figures 4 and 5, which show the adjusted prediction of GAD-7 and PHQ-9 scores after considering the variables included in the mixed-effects models.

#### Sensitivity Analysis

The sensitivity analysis was conducted to test whether the model and covariance structures were mis-specified. We began by checking the correlation and covariance structures (in both statistical parameter and visual manner). Initially, the correlation structure (Multimedia Appendix 12) suggested an unstructured correlation, but then we specified all other major types of covariance structures: independent, unstructured, exchangeable, identity, and autoregressive. We also used both the maximum likelihood and restricted maximum likelihood methods to estimate the model parameters. Although the results of the descriptive statistics (Multimedia Appendix 13) suggested that no sociodemographic variables were significantly associated with each outcome measure, the GAD-7 and PHQ-9, we included them in the models. We found that only models with exchangeable structure fit the data and remained constant both including and excluding sociodemographics.

## Results

### Participant Characteristics

As can be seen in Table 1, the average age of the participants was 34.17 (SD 10.50) years. Of the 103 participants enrolled in this study, most were female (n=66, 64.1%), resided in city and metropolitan areas (n=75, 72.8%), were neither Aboriginal nor Torres Strait Islanders (n=100, 97.1%), were heterosexual (n=77, 74.8%), were in a relationship (n=69, 67%), and earned an income of Aus ≥$20,000 (US $13,533.30; n=77, 74.8%). Most had completed tertiary education (n=58, 56.3%), were employed full-time or part-time (n=58, 56.3%), and reported being dissatisfied with their sleep patterns. Most participants (76/103, 73.8%) consumed alcohol infrequently, had never used illicit drugs, and had accessed both physically and mentally focused health services. There were no significant differences among the 3 intervention groups in any of these sociodemographic variables (all *P*>.05). Further demographic variable information is presented in Multimedia Appendix 13.

**Table 1:** The sociodemographic and clinical characteristics of the sample at baseline by intervention condition (n=103).

Variable		Overall	DMH^a^ (n=42)	DMH+LI^b^ (n=29)	DMH+HI^c^ (n=32)	*P* value	
Age (years), mean (SD)		34.17 (10.5)	35.17 (12.5)	31.55 (7.8)	35.25 (9.4)	.29^d^	
**Sex,** **n (%)**							.60
	Male	34 (33)	11 (26.2)	11 (37.9)	12 (37.5)		
	Female	66 (64.1)	29 (69)	17 (58.6)	20 (62.5)		
	Other	3 (2.9)	2 (4.8)	1 (3.4)	0 (0)		
**Residential area,** **n (%)**							.77
	City or metropolitan	75 (72.8)	32 (76.2)	21 (72.4)	22 (68.8)		
	Regional or rural or remote	28 (27.2)	10 (23.8)	8 (27.6)	10 (31.3)		
**Aboriginal and Torres Strait Islander, n (%)**							.49
	Non–Aboriginal and Torres Strait Islander	100 (97.1)	41 (97.6)	28 (96.6)	31 (96.9)		
	Aboriginal and Torres Strait Islander	2 (1.9)	1 (2.4)	0 (0)	1 (3.1)		
	Prefer not to disclose	1 (1)	0 (0)	1 (3.4)	0 (0)		
**Sexual orientation,** **n (%)**							.81
	Heterosexual	77 (74.8)	30 (71.4)	23 (79.3)	24 (75)		
	LGBTQ^e^	17 (16.5)	7 (16.7)	5 (17.2)	5 (15.6)		
	Another or rather not say	9 (8.7)	5 (11.9)	1 (3.4)	3 (9.4)		
**Educational level, n (%)**							.35
	High school	14 (13.6)	4 (9.5)	4 (13.8)	6 (18.8)		
	Certificate level	31 (30.1)	11 (26.2)	10 (34.5)	10 (31.3)		
	Undergraduate degree	46 (44.7)	23 (54.8)	9 (31)	14 (43.8)		
	Postgraduate degree	12 (11.7)	4 (9.5)	6 (20.7)	2 (6.3)		
Religion^f^, mean (SD)		2.04 (2.7)	2.36 (2.9)	1.38 (1.8)	2.22 (3.1)	.30^d^	
**Relationship status,** **n (%)**							.96
	Single	34 (33)	14 (33.3)	10 (34.5)	10 (31.3)		
	In a relationship	69 (67)	28 (66.7)	19 (65.5)	22 (68.8)		
**Employment status,** **n (%)**							.59
	Full-time	42 (40.8)	14 (33.3)	12 (41.4)	16 (50)		
	Part-time	16 (15.5)	7 (16.7)	6 (20.7)	3 (9.4)		
	Carer or volunteer support or retired	8 (7.8)	2 (4.8)	3 (10.3)	3 (9.4)		
	Studying	14 (13.6)	6 (14.3)	3 (10.3)	5 (15.6)		
	Unemployed	11 (10.7)	5 (11.9)	4 (13)	2 (6.3)		
	Other	12 (11.7)	8 (19)	1 (3)	3 (9.4)		
**Annual income (Aus $ [US $]),** **n (%)**							.91
	0-19,999 (0-13,532.70)	15 (14.6)	7 (16.7)	4 (13.8)	4 (12.5)		
	20,000-80,000 (13,533.30-54,133.40)	48 (46.6)	21 (50)	14 (48.3)	13 (40.6)		
	≥80,000 (54,133.40)	29 (28.2)	11 (26.2)	7 (24.1)	11 (34.4)		
	Prefer not to disclose	11 (10.7)	3 (7.1)	4 (13.8)	4 (12.5)		
Number of residents they live with, mean (SD)		1.67 (1.2)	1.57 (1.1)	1.93 (1.5)	1.56 (1.2)	.41^d^	

^a^DMH: digital mental health.

^b^LI: low-intensity therapist assistance.

^c^HI: high-intensity therapist assistance.

^d^*P* values based on ANOVA; the remaining were based on the chi-square test.

^e^LGBTQ: lesbian, gay, bisexual, transgender, queer.

^f^Religion was scored from 0 to 10, with higher scores reflecting higher levels of religion.

### Primary Outcomes

Table 2 displays the results of the outcome measures across assessment time points by intervention condition (DMH intervention program only and low- and high-intensity therapist assistance). There were significant changes in most of these outcomes over time. Of note, all 3 intervention conditions demonstrated strong and significant treatment effect changes on the 2 primary outcomes (GAD-7 and PHQ-9 scores; all *P*<.05) and one of the secondary outcomes (mental health rating; all *P*<.05), with absolute values of the Cohen *d* ranging from 0.82 to 1.79.

The remaining secondary outcomes achieved only a small to moderate effect size change, although not all of them were significant (all *P*>.05). No significant changes were observed in the quality-of-life health rating, sleeping time, and physical health ratings across time points in the low-intensity therapist assistance condition and in social support and physical health ratings in the DMH intervention program–only condition (all *P*>.05). A moderate effect size was also found for social support in the high-intensity therapist-assisted condition.

Mixed-effects regression analyses were then performed using the time points and intervention conditions as main effects. Given that there were no sociodemographic variables significantly associated with intervention conditions from the unadjusted analysis (Table 1 and Multimedia Appendix 13), only baseline outcomes (GAD-7 and PHQ-9 scores) were entered into the model for addressing the regression-to-the-mean issue.

In the fixed-effects component, at week 0 and in the DMH intervention program–only condition, the mean GAD-7 and PHQ-9 scores were 3.29 and 3.22, respectively. At week 3, mean GAD-7 and PHQ-9 scores significantly decreased by 3.54 and 4.38 compared with the baseline (all *P*<.001), respectively. At weeks 6, 9, and 21, GAD-7 scores significantly decreased by at least 6 points, whereas PHQ-9 scores decreased by at least 7 points compared with the baseline (all *P*<.001). There were no significant differences in GAD-7 and PHQ-9 score changes between the DMH intervention program–only and the low- or high-intensity therapist-assisted conditions (all *P*>.05).

For the random effects, the overall sample mean scores on the GAD-7 and PHQ-9 were 3.29 and 3.22, respectively, as mentioned previously. Each participant had an average SD of 1.56 and 1.79, respectively, around that overall sample mean, whereas each time point measurement also showed an SD of 1.56 and 1.79 around each participant’s mean, suggesting a considerable effect. The positive correlation coefficient of 0.99 to 1.00 between time point and intercept indicated that participant baseline outcome mean scores that were higher than the intercept (or the sample mean) were more likely to decrease compared with lower mean values. Both mixed-effects models of the GAD-7 and PHQ-9 fit the data based on the fit criteria and when compared with conventional linear regression and random-intercept models (Table 3). The model fit criteria in Table 3 suggest that our model was appropriate.

Figures 2 and 3 show the effect of the DMH intervention program on the change in the 2 primary outcome measures, GAD-7 and PHQ-9, respectively. Overall, all 3 intervention groups showed a significant decrease in these outcomes over time (*P*<.001). The rapid decrease from the preintervention time point (week 0) to during the intervention (weeks 3 and 6) and the postintervention time point (week 9) was observed for both outcome measures. However, there was no change after the postintervention time point for PHQ-9 scores, whereas GAD-7 scores continued to decrease after week 9, although slowly. There was no interaction effect between time point and intervention condition, suggesting the same treatment effect on the outcome measures.

The reliable change index was calculated as 5.06 for pre- and post- and 4.53 for pre- and follow-up treatment, respectively, for anxiety (GAD-7 Cronbach α at the preintervention, postintervention, and follow-up time points was .87, .88, and .93, respectively, and baseline SD was 5.16) and as 6.02 for pre- and post- and 4.55 for pre- and follow-up treatment, respectively, for depression (PHQ-9 Cronbach α at the preintervention, postintervention, and follow-up time points was .81, .84, and .99, respectively, and baseline SD was 5.19). Figures 4 and 5 show the change in GAD-7 and PHQ-9 scores from baseline (time 0) to the postintervention time point (time 1; Figures 4A and 5A) and the 3-month follow-up (time 2; Figures 4B and 5B). A total of 42% (31/74) and 49% (34/69) of the participants experienced reliable and clinically significant improvements at time 1 on the GAD-7 and PHQ-9, respectively, and 52% (32/62) and 61% (39/64) experienced reliable and clinically significant improvements at time 2 on the GAD-7 and PHQ-9, respectively (Multimedia Appendix 14). Details of the participants showing individual reliable change above the threshold after considering typical errors in the GAD-7 and PHQ-9 are presented in Multimedia Appendices 15 and 16. Changes in GAD-7 and PHQ-9 scores between time points across intervention conditions are shown in Multimedia Appendices 6-11.

**Table 2:** Change in intervention outcomes over time (n=103).

Variable and condition		Preintervention time point (week 0), mean (SD)	Postintervention time point (week 9), mean (SD)	Follow-up (week 21), mean (SD)	η^2a^	*P* value^b^	Cohen *d* (week 0 vs week 9)	*P* value^c^	Cohen *d* (week 0 vs week 21)	*P* value^d^
**GAD-7^e^**										
	DMH^f^ (n=42)	10.98 (5.32)	3.88 (4.68)	3.71 (4.50)	0.40	<.001	1.43	<.001^g^	1.49	<.001^g^
	DMH+LI^h^ (n=29)	12.03 (4.95)	4.28 (5.40)	3.86 (6.48)	0.45^d^	<.001	1.52	<.001^g^	1.44	<.001^g^
	DMH+HI^i^ (n=32)	11.47 (5.25)	7.50 (4.22)	6.50 (5.08)	0.28	<.001	0.82	.001	0.90	.02^g^
**PHQ-9^j^**										
	DMH (n=42)	13.14 (4.64)	4.62 (5.00)	4.62 (5.58)	0.45^d^	<.001	1.79	<.001^g^	1.68	<.001^g^
	DMH+LI (n=29)	12.45 (5.72)	4.55 (6.31)	4.52 (7.41)	0.45^d^	<.001	1.34	<.001^g^	1.22	<.001^g^
	DMH+HI (n=32)	13.03 (5.51)	6.95 (3.96)	6.86 (4.19)	0.38^d^	<.001	1.23	<.001	1.13	.01^g^
**Quality-of-life health rating**										
	DMH (n=42)	3.43 (0.99)	4.00 (0.86)	4.12 (0.83)	0.23^d^	<.001	−0.60^h,^^i^	.004	−0.73^d,^^i^	<.001
	DMH+LI (n=29)	3.62 (1.21)	4.06 (1.20)	4.07 (1.54)	0.13	.12	−0.36^l^	.06	−0.33^l^	.07
	DMH+HI (n=32)	3.53 (1.02)	4.18 (0.80)	4.43 (0.94)	0.32^h^	.001	−0.70^d^	<.001	−0.85^j^	.01
**Quality-of-life utility index**										
	DMH (n=42)	0.57 (0.18)	0.61 (0.18)	0.68 (0.18)	0.21^h^	.003	−0.24	.32^g^	−0.61	<.001^g^
	DMH+LI (n=29)	0.51 (0.21)	0.64 (0.25)	0.60 (0.28)	0.22^j^	.03	−0.58^h^	.001	−0.35^l^	.09
	DMH+HI (n=32)	0.56 (0.23)	0.70 (0.17)	0.67 (0.19)	0.27^h^	.005	−0.65^h^	.004	−0.46	.10
**Social support**										
	DMH (n=42)	2.93 (0.87)	3.18 (0.98)	3.08 (1.06)	0.03	.50	−0.27^i^	.08	−0.16^i^	.30
	DMH+LI (n=29)	2.86 (0.99)	3.12 (1.27)	3.20 (1.32)	0.02	.78	−0.23	.74	−0.30	.74
	DMH+HI (n=32)	2.97 (1.09)	3.27 (1.20)	3.00 (1.04)	0.15	.07	−0.27	.18	−0.03	.92
**Sleep hour**										
	DMH (n=42)	405.83 (70.81)	434.21 (69.79)	440.52 (56.87)	0.19^h^	.005	−0.40	.007^g^	−0.52	.01^g^
	DMH+LI (n=29)	442.24 (78.42)	435.88 (75.09)	463.93 (98.53)	0.06	.43	0.08	.62	−0.24	.06
	DMH+HI (n=32)	425.31 (79.64)	437.59 (61.09)	431.79 (59.47)	0.07	.31	−0.17	.15	−0.08	.79
**Physical health rating**										
	DMH (n=42)	3.50 (0.99)	3.57 (0.88)	4.00 (0.82)	0.08	.12	−0.07	.91^g^	−0.53	.08^g^
	DMH+LI (n=29)	3.38 (1.27)	3.41 (1.37)	3.71 (1.33)	0.06	.47	0.02	.67	−0.25	.22
	DMH+HI (n=32)	3.13 (1.18)	3.64 (1.05)	3.57 (1.02)	0.36^d^	<.001	−0.45^h^	.001	−0.37^h^	.005
**Mental health rating**										
	DMH (n=42)	2.62 (0.66)	3.64 (0.91)	3.68 (0.80)	0.45^d^	<.001	−1.32	<.001^g^	−1.45	<.001^g^
	DMH+LI (n=29)	2.45 (0.74)	3.29 (0.99)	3.29 (1.54)	0.31^h^	.005	−1.00^d^	<.001	−0.76^j^	.02
	DMH+HI (n=32)	2.56 (0.84)	3.55 (0.91)	3.57 (1.02)	0.38^d^	<.001	−1.13^d^	<.001	−1.06^j^	.03

^a^Effect size was based on repeated measure.

^b^*P* value was based on repeated measure ANOVA.

^c^*P* value was based on one-tailed paired *t* test to compare outcome value at week 0 and week 9.

^d^*P* value was based on one-tailed paired *t* test to compare outcome value at week 0 and week 21.

^e^GAD-7: 7-item Generalized Anxiety Disorder Scale.

^f^DMH: digital mental health.

^g^*P* value was based on one-tailed Wilcoxon signed rank test.

^h^LI: low-intensity therapist assistance.

^i^HI: high-intensity therapist assistance.

^j^PHQ-9: 9-item Patient Health Questionnaire.

**Table 3:** Mixed-effects linear regression analysis of the effect of intervention condition on 7-item Generalized Anxiety Disorder Scale (GAD-7) and 9-item Patient Health Questionnaire (PHQ-9) improvement.

Variable				GAD-7 regression coefficient (95% CI)		*P* value		PHQ-9 regression coefficient (95% CI)		*P* value
**Fixed effects**										
	Constant			3.29 (1.68 to 4.91)		<.001		3.22 (1.25 to 5.20)		.001
	**Time point**									
		Week 0	Reference		N/A^a^		Reference		N/A	
		Week 3	−3.54 (−4.52 to −2.56)		<.001		−4.38 (−5.40 to −3.35)		<.001	
		Week 6	−6.02 (−7.03 to −5.01)		<.001		−7.13 (−8.18 to −6.08)		<.001	
		Week 9	−6.62 (−7.63 to −5.61)		<.001		−7.88 (−8.94 to −6.83)		<.001	
		Week 21	−7.10 (−8.14 to −6.06)		<.001		−7.91 (−8.99 to −6.93)		<.001	
	**Condition**									
		DMH^b^	Reference		N/A		Reference		N/A	
		DMH+LI^c^	−0.31 (−1.73 to 1.12)		.67		−0.26 (−1.84 to 1.31)		.74	
		DMH+HI^d^	0.88 (−0.54 to 2.29)		.26		0.55 (−1.00 to 2.10)		.49	
		Baseline^e^	0.70 (0.58 to 0.81)		<.001		0.74 (0.62 to 0.87)		<.001	
**Random effects**										
	SD (time)			1.56 (1.27 to 1.93)		N/A		1.79 (1.47 to 2.18)		N/A
	Correlation (time and intercept)			1.00 (−1 to 1)		N/A		0.99 (−1 to 1)		N/A
	SD (residual)			3.40 (3.16 to 3.66)		N/A		3.50 (3.25 to 3.77)		N/A
**Model fit**										
	−2Log-likelihood			2663.07		N/A		2707.84		N/A
	Wald chi-square (*df*=7)			396.4		<.001		440.8		<.001
	Chi-square (*df*=2; LRT^f^ vs linear model)^g^			84.4		<.001		114.1		<.001
	Chi-square (*df*=1; LRT vs random-intercept model)^h^			44.8		<.001		55.4		<.001

^a^N/A: not applicable.

^b^DMH: digital mental health.

^c^LI: low-intensity therapist assistance.

^d^HI: high-intensity therapist assistance.

^e^GAD-7 baseline for GAD-7 as outcome and PHQ-9 baseline for PHQ-9 as outcome.

^f^LRT: likelihood ratio test.

^g^Chi-square and *P* value of the LRT to compare the (current) random-intercept and random-slope model with the linear model.

^h^Chi-square and *P* value of the LRT to compare the (current) random-intercept and random-slope model with the random-intercept model.

### Diagnostic Status

At the preintervention time point, all except 1 (1%) out of 103 participants were assessed as having clinical anxiety or depression. The most common diagnosis was major depressive disorder comorbid with at least one anxiety disorder (57/103, 55.3%) followed by major depressive disorder (27/103, 26.2%), with 12.6% (13/103) of the participants also meeting the criteria for more than one anxiety disorder. At the postintervention time point, 63.1% (65/103) of the participants attended the diagnostic assessment, with 68% (44/65) of them no longer meeting the criteria for a Diagnostic and Statistical Manual of Mental Disorders [51] diagnosis. At the 3-month follow-up, 47.6% (49/103) of the participants attended the diagnostic assessment, with 69% (34/49) of them no longer meeting any diagnostic criteria. Further information on the diagnostic data gathered at the postintervention time point and the 3-month follow-up is presented in Multimedia Appendix 17, with preintervention data included for comparison.

### Program Use

The mean number of log-ins to the Life Flex program for the sample of 103 participants was 24.41 (SD 23.53), and the average total number of pages viewed per participant was 132.54 (SD 100.01). A between-group analysis of variance was completed, with nonsignificant interaction effects found for total program log-ins (*F*_2,100_=1.81; *P*=.17), pages viewed (*F*_2,100_=1.84; *P*=.16), and the percentage of the program completed (*F*_2,100_=1.93; *P*=.15). The number of program log-ins, pages viewed, and percentage of program completion for the total sample (n=103) and the 3 treatment conditions are presented in Table 4.

**Table 4:** Program use (n=103).

	Log-ins, mean (SD)	Pages viewed, mean (SD)	Percentage of the program completed, mean (SD)
Total sample	24.4 (23.5)	132.5 (100.01)	64.6 (35.6)
DMH^a^ (n=42)	28.7 (21.4)	146.9 (81.70)	72.5 (30.9)
DMH+LI^b^ (n=29)	17.9 (19.1)	102.9 (77.30)	57.4 (38.2)
DMH+HI^c^ (n=32)	24.7 (28.6)	140.6 (132.3)	60.8 (34.7)

^a^DMH: digital mental health.

^b^LI: low-intensity therapist assistance.

^c^HI: high-intensity therapist assistance.

### Low- and High-Intensity Therapist-Assisted Sessions

Throughout the clinical trial, all participants in the low- (29/103, 28.2%) and high-intensity (32/103, 31.1%) therapist assistance conditions were offered 7 video chat sessions of therapist assistance adjunctive to the Life Flex program. Of the 29 participants randomized to receive low-intensity therapist assistance, 7 (24%) did not engage in or attend any sessions. Among the 22 low-intensity condition participants who did attend the video chat sessions, the mean number of sessions attended was 5.4 (SD 2.36), and the mean total therapist time per participant in the low-intensity therapist-assisted condition was 82 (SD 36.36) minutes. Of the 32 participants randomized to receive high-intensity therapist assistance, 5 (16%) did not engage in or attend any sessions. Among the 27 participants who did attend the video chat sessions, the mean number of sessions attended was 5.4 (SD 2.0), with a mean of 273 (SD 111.14) minutes of therapist assistance provided per participant.

### Program Usability

At week 3, participants were asked to rate the usability of the Life Flex program by completing the SUS [46]. In total, 87.4% (90/103) of the participants completed the questionnaire, providing a total usability score of 72.61/100 (SD 6.5), which is within an acceptable range.

### Treatment Satisfaction

At the postintervention time point, participants were asked to rate how satisfied they were with the Life Flex program on a 4-point scale ranging from 1=poor to 4=excellent. In total, 65% (67/103) of the participants provided treatment satisfaction ratings, with scores of ≥75% indicative of treatment acceptability. In total, 85% (57/67) of the participants rated the overall quality of treatment delivered by Life Flex as good to excellent, with 84% (56/67) of the participants reporting that they were very satisfied with the program. Additional participant satisfaction ratings are shown in Multimedia Appendix 18. Participants were also invited to provide comments on the most and least preferred aspects of the Life Flex program. The most frequently cited preferred aspects of the program included the ease of program navigation, program information (particularly learning about neuroplasticity), and the variety of useful strategies to choose from. The commonly cited least preferred aspects of the program included issues with mobile navigation and compatibility, not having videos with subtitles, content being too wordy at times, and difficulty finding time in a busy schedule to complete the program.

## Discussion

### Principal Findings

The primary objective of this adaptive clinical trial was to evaluate the efficacy of various support intensities of a transdiagnostic biopsychosocial DMH intervention program, Life Flex, for anxiety or depression in adults. This study aimed to investigate the efficacy of an adaptive intervention design by testing whether therapist assistance (low and high intensity) in the form of video chat support produced significant clinical outcomes compared with a self-help DMH intervention program only. In the sample of 103 participants, we found no statistically significant difference between the participants who were stepped up to receive therapist assistance and those who remained in the DMH intervention program–only condition for outcomes of anxiety and depression. The participants in all 3 treatment conditions demonstrated significant improvement in anxiety and depression over the course of the intervention, with a large treatment effect size change. Quality of life (scores on the health-related quality-of-life utility index) improved across all 3 treatment conditions. This section highlights some of the benefits and challenges of adaptive intervention research designs, with implications for the stepped-care models discussed.

As this trial used an adaptive intervention design, the results indicate that participants who continued in the Life Flex program–only condition did well. Regarding those participants who met the stepped-care criteria of the study, it could also be argued that they did well after having their treatment program augmented with therapist assistance. It could be speculated that, without the adaptive design, participants who did not engage with the program or those with moderate-to-high symptom severity may have dropped out completely from the Life Flex program–only condition.

In step 1 of the adaptive trial, all eligible participants (n=113) were given access to the preintervention questionnaire. At week 3, a total of 64.6% (73/113) of the participants met the criteria to have their treatment program augmented with therapist assistance. For 58% (42/73) of these participants, the stepped-care rule of no program engagement was met, followed by 26% (19/73) who met the no-engagement and noncompletion of scheduled assessment rule, 7% (5/73) who met symptom severity criteria, and 10% (7/73) who met both the symptom severity and no-program-engagement criteria. These results are consistent with previous literature investigating engagement rates of self-help DMH interventions that have demonstrated high attrition and low completion rates in the absence of stepped care [17,22,55].

The clinical trial presented an opportunity to capture low engagement early on, which meant that most participants allocated to low-intensity (29/36, 81%) and high-intensity (32/37, 86%) therapist assistance had the opportunity to access therapist assistance. This is important given the literature demonstrating the relationship between engagement and positive symptom outcomes in DMH intervention research [56,57]. Other researchers have evaluated the cumulative findings of 4 randomized controlled trial studies to assess the timing and magnitude of symptom improvement in internet-delivered transdiagnostic treatment for anxiety and depression [58]. The authors concluded that the first month of treatment is the most important for determining symptom improvement and overall participant outcomes. Therefore, research that incorporates an adaptive intervention design has the potential to positively influence the trajectory of symptom change and engagement promptly as participants can be stepped up to receive more intensive treatment based on nonengagement or symptom severity.

The large treatment effect size change found for the DMH intervention program–only condition in this study is consistent with an earlier single-group evaluation demonstrating the preliminary effectiveness of a fully automated self-help version of the Life Flex program [59]. In both this study and previous research [59], large treatment effect sizes were found for the Life Flex program–only participants at the postintervention time point and 3-month follow-up for comorbid anxiety and depression. Therefore, the underlying mechanisms of symptom change can be examined from the perspective of the effectiveness of the Life Flex program itself. For instance, other researchers [60] have hypothesized that therapist assistance may be less important in the context of a credible, high-quality DMH intervention program that is engaging for participants. The acceptability and satisfaction with the Life Flex program in a previous research study [59] was high, as was the case in this study. The Life Flex program extends beyond traditional transdiagnostic intervention programs through the addition of neuroplasticity principles, strategies designed to increase biological flexibility (ie, allostasis), and integration of positive psychology strategies (ie, increasing positive affect), which may enhance treatment effectiveness. It is also possible that the psychoeducation and accompanying brain plasticity strategies delivered within the Life Flex program can enhance self-motivation for change. The findings of previous research [59] and this study suggest that, in the absence of therapist assistance, the dissemination of a fully automated self-guided transdiagnostic biopsychosocial DMH intervention program is a viable and accessible treatment service delivery option for those who prefer self-help models.

The finding of no significant difference among the 3 treatment conditions could also be explained by the nature of the clinical trial design. All participants underwent prescreening (semistructured phone interview and video chat diagnostic assessment), followed by postintervention and 3-month follow-up video chat diagnostic assessment interviews with provisional or generally registered psychologists. This contact with research personnel may have been therapeutic for participants. Previous research evaluating the timing and magnitude of symptom improvement found participants to improve following an initial assessment call, with authors concluding that even brief therapeutic contact can influence symptom outcomes in clinical trials [58]. All participants also received automated emails throughout the trial to encourage progress and maintain engagement, with the same duration of access to the Life Flex program provided to all participants. The Life Flex program content was accessed through a scheduled release design, meaning that expectations for program completion were clear and consistent across all treatment conditions. Previous research [31,61,62] has also proposed that increasing therapist contact beyond a certain threshold may have limited gains, which may partly explain the lack of difference between the low- and high-intensity therapist assistance offered in this study.

It is also important to consider the influence of participant self-selection bias [63] and the possibility of participant influence on stepped-care eligibility in the clinical trial. Self-selection bias in clinical trials is common and refers to the phenomenon of disproportionate self-selection into a study [63]. The theory of planned behavior [64] presents a useful contextual framework to better understand the influence of participant self-selection bias in clinical trials. The theory of planned behavior states that decision-making resulting in planned behaviors is a result of the complex interplay among a person’s beliefs, attitudes, subjective norms, and perceived control. This trial collected preference data from all participants before the implementation of the adaptive intervention design. Within the theory of planned behavior, participant preferences could be reflective of the attitude component, and their ability to influence eligibility for stepped-care criteria could reflect perceived control [64]. In this study, it could be hypothesized that a participant’s behavioral intentions to enroll in a clinical trial only to not engage with the program content but complete the scheduled assessments (42/73, 58%) represents a planned desire to have their treatment program stepped up to work with a therapist in either the low- or high-intensity therapist-assisted condition. Most participants eligible for stepped care (49/73, 67%) were contactable and able to be randomized and attended either low- or high-intensity therapist-assisted sessions, progressing from nonengagement to engagement with the Life Flex program.

Closer examination of the available first treatment preferences of the participants who met the stepped-care criteria (63/73, 86%) indicates that 68% (43/63) preferred high-intensity therapist assistance, 17% (11/63) preferred low-intensity therapist assistance, and only 14% (9/63) preferred the Life Flex program only. The preferences of the participants who did not meet the stepped-care criteria were also examined. Of these participants, 45% (18/40) had a first treatment preference for high-intensity therapist assistance, 35% (14/40) had a preference for low-intensity therapist assistance, and 20% (8/40) had a preference for the Life Flex program only. The preference data across the entire sample indicate a strong preference among participants for therapist assistance (86/103, 83.5% first treatment preference for therapist assistance compared with 17/103, 16.5% preference for the Life Flex program only). This finding is similar to that of previous research [65], which found that 73% of the participants allocated to self-help desired contact with a therapist for the email support on offer. What may be influential in this study is the climate in which the clinical trial was conducted. Recruitment for the trial commenced in November 2020, several months into the COVID-19 pandemic, a time when demand for mental health services substantially increased [66,67] and there were long waiting lists for traditional psychological services [68,69]. Therefore, it may be possible that a higher-than-usual proportion of the participants in the clinical trial offering a DMH intervention self-selected to participate as a possible means to gain access to a therapist. Although participant preference data were collected, the personal choices of the participants to be stepped up to receive therapist assistance were not considered. Future studies should investigate the potential impacts of incorporating participant preferences into decisions regarding stepped-care rules.

Despite preferences, participants who remained in the Life Flex program–only condition did just as well with regard to symptom reduction of primary outcomes (anxiety and depression) as participants who were stepped up to receive therapist assistance. The health-related quality-of-life utility index scores also indicated significant improvements in quality of life across all 3 treatment conditions. Given that there were no differences among the 3 conditions, the Life Flex program by itself could be the most accessible and cost-effective treatment option. The Life Flex program–only condition also had the highest percentage of program completion of the 3 treatment conditions at 72% compared with 57% for low-intensity therapist assistance and 60% for high-intensity therapist assistance. Therefore, it could also be speculated that participants may have relied on their video chat sessions with their therapist for delivery of program content, reducing engagement with the Life Flex program itself.

Participants who were stepped up had their treatment program augmented with therapist assistance delivered via video chat technology. In both the low- and high-intensity therapist assistance conditions, participants received individualized guidance, with a key component of therapist assistance involving reinforcement and motivation enhancement. Therefore, it would be beneficial to further investigate self-efficacy and motivation as predictors of engagement and attrition (as is planned in a subsequent paper by the authors) to better understand who is most suited for self-help and who may benefit from therapist assistance within DMH adaptive intervention designs. Research designed to improve the implementation of stepped care through contextual behavioral science recommends that the “match-mismatch” design be applied to stepped-care research to enhance understanding of treatment intensity and dose based on participant-specific characteristics [25]. Other studies are demonstrating the importance of increasing expectations among possible consumers of DMH treatments through education and marketing, which may increase mental health literacy among consumers regarding the various evidence-based treatment options available [70]. Investigating the effectiveness of other research designs and evaluating participant attitudes and perceived therapeutic needs may reduce participant bias and further enhance referral pathways within stepped-care treatment models.

### Limitations

This study did not control for participant contact with the research personnel. This lack of control means that we cannot observe whether the symptom changes were due to the treatment delivered or other extraneous factors such as time and contact with research personnel within a clinical trial. As this study involved 3 assessment time points for the Life Flex program–only participants, future research is required to replicate and evaluate the study findings taking this into consideration. This study also did not have a control condition for the nonresponders, meaning that we are unable to determine whether these participants would have disengaged and whether they improved relative to receiving therapist assistance. Participants who engaged or showed symptom improvement were also only given the option of continuing in the Life Flex program–only condition. Therefore, the possible impact of assigning a therapist to the participants who engaged or showed symptom improvement cannot be determined within this study design.

As the trial excluded participants with severe suicidal ideation, this study cannot present the baseline magnitude of depressive severity for individuals who presented with active suicidal ideation. Therefore, trial results are limited to participants experiencing depressive symptoms without active suicidal ideation, which potentially limits the generalizability of the results as it is not representative of conditions in routine clinical practice. Masking of participants and therapists to treatment condition was not possible, introducing a possible risk of bias; however, therapists did not have access to participant preference data, nor were they aware of the study’s objectives. This study also did not ask participants about their previous treatment experiences, which may have affected their preferences and mental health literacy. The study was also conducted during the global COVID-19 pandemic, which may have posed several challenges, including participants’ self-selection into the clinical trial in the hope of accessing therapist assistance and their commitment to the trial being negatively affected, as well as their symptoms of anxiety or depression because of the considerable uncertainty present in the community throughout data collection. However, the diagnostic status results at the postintervention time point and 3-month follow-up are promising of considerable symptom changes.

### Implications

Despite these identified limitations, this study has several important implications. The study shows respect for the evidence base and clinical guidelines of stepped-care models [71-73] and considers the role that participant preferences may play within adaptive DMH intervention designs. Although our findings indicate that an adaptive DMH intervention design may reduce therapist time by offering a DMH intervention program without support as a first step, we offer some considerations for extending these findings beyond a trial setting. The participant preference data showed a clear preference for working with a therapist adjunctive to the DMH intervention program. Preferences appear to be important to participants. However, no differences were found among the 3 treatment conditions in terms of outcomes. Therefore, preferences may have implications for clinical decision-making in stepped-care models when it comes to considering factors influencing engagement.

Given the nature of a clinical trial, the study used strict criteria to determine stepped-care eligibility and a randomized approach to participant allocation to low- and high-intensity therapist assistance. This contrasts with stepped-care decision-making typically used outside research contexts, which heavily relies on clinical judgment [74] and can vary substantially among clinicians, leading to less standardization and clarity in decision-making. The treatment was also implemented in a way that maintained fidelity to the treatment model, with fidelity procedures outside of daily supervision incorporating quantitative monitoring used for the therapists conducting assessments and delivering therapist assistance. The treatment duration was consistent across all 3 treatment conditions, and although only 1% (1/103) of the participants were assessed before the intervention as having subthreshold major depressive disorder, the study was inclusive in recruitment as it did not exclude participants with subthreshold symptomatology, as is commonly the case in clinical trial research.

### Conclusions

This study adds to the existing body of literature on stepped-care models and advances the research field of adaptive intervention designs and therapist assistance delivered via video chat technology. Significant improvements in the primary outcomes of anxiety and depression were observed across all 3 treatment conditions, with high levels of treatment acceptability and program completion reported. Adaptive research designs that aim to improve the efficiency and efficacy of DMH interventions while considering participant characteristics of nonengagement and symptom severity present both opportunities and challenges. Although these findings indicate that therapist assistance was no more effective than the DMH intervention program alone for reducing symptoms of anxiety or depression, the data highlight the potential influence of participant selection bias and participant preferences within stepped-care treatment models. Under the caveat that all participants had therapist contact for the diagnostic assessments, the findings present evidence that transdiagnostic DMH interventions can be effective with or without therapist assistance; however, further research is needed to consolidate conclusions.

## Appendix

Multimedia Appendix 1 CONSORT-EHEALTH (Consolidated Standards of Reporting Trials of Electronic and Mobile Health Applications and Online Telehealth) checklist.

Multimedia Appendix 2 Life Flex program modules.

Multimedia Appendix 3 Participant preferences.

Multimedia Appendix 4 Descriptive of outcome at baseline by conditions.

Multimedia Appendix 5 Change in intervention outcomes over time.

Multimedia Appendix 6 Reliable and clinically significant change in 7-item Generalized Anxiety Disorder Scale (GAD-7) score among digital mental health intervention–only program participants.

Multimedia Appendix 7 Reliable and clinically significant change in 7-item Generalized Anxiety Disorder Scale (GAD-7) score among low-intensity therapist-assistance participants.

Multimedia Appendix 8 Reliable and clinically significant change in 7-item Generalized Anxiety Disorder Scale (GAD-7) score among high-intensity therapist-assistance participants.

Multimedia Appendix 9 Reliable and clinically significant change in 9-item Patient Health Questionnaire (PHQ-9) score among digital mental health intervention–only program participants.

Multimedia Appendix 10 Reliable and clinically significant change in 9-item Patient Health Questionnaire (PHQ-9) score among low-intensity therapist-assistance participants.

Multimedia Appendix 11 Reliable and clinically significant change in 9-item Patient Health Questionnaire (PHQ-9) score among high-intensity therapist-assistance participants.

Multimedia Appendix 12 Correlations among outcomes and repeated measures.

Multimedia Appendix 13 Remainder of the sociodemographic and clinical characteristics of the sample at baseline by intervention conditions.

Multimedia Appendix 14 Reliable and clinically significant change in intervention primary outcomes over times across intervention conditions.

Multimedia Appendix 15 Distribution of the individual 7-item Generalized Anxiety Disorder (GAD-7) change by reliable change status in different time points.

Multimedia Appendix 16 Distribution of the individual 9-item Patient Health Questionnaire (PHQ-9) change by reliable change status in different time points.

Multimedia Appendix 17 Frequency of each disorder at preintervention, postintervention, and follow-up time points.

Multimedia Appendix 18 Treatment satisfaction ratings.

## Abbreviations

ANZCTR: Australian New Zealand Clinical Trials Registry
AQol-4D: Assessment of Quality of Life
CBT: cognitive behavioral therapy
CMOTS: Client Motivation for Therapy Scale
CONSORT-EHEALTH: Consolidated Standards of Reporting Trials of Electronic and Mobile Health Applications and Online Telehealth
DMH: digital mental health
GAD-7: 7-item Generalized Anxiety Disorder Scale
PHQ-9: 9-item Patient Health Questionnaire
SUS: System Usability Scale
WAI-S: Working Alliance Inventory–Short

## Data Availability

The raw, nonidentifiable data supporting the conclusions of this study will be made available by the corresponding author following a request and an approval process by the Federation University human research ethics committee for access to these data (as per the study’s approved ethics application).
