# Supplementary material for: Efficacy of a Digital Mental Health Biopsychosocial Transdiagnostic Intervention With or Without Therapist Assistance for Adults With Anxiety and Depression: Adaptive Randomized Controlled Trial
Source: J Med Internet Res. 2023 Jun 12;25:e45135. doi: 10.2196/45135 (PMC10337336; doi:10.2196/45135)
Supplement: Multimedia Appendix 2 [file jmir_v25i1e45135_app2.docx]

## Appendix 2

Table S1. Life Flex Program Modules

| Name of Module | Module Overview |
| --- | --- |
| Introduction | Provides a rationale for the Life Flex program, key theories and information about anxiety and depression, as well as exploration of factors contributing to motivation for change  Activities include self-monitoring of anxiety and depressive symptoms and practice of either ‘Successful Events’ or ‘Positive statements’ activity. Participants are also presented with a resource for suicidal thoughts (if applicable) and keeping safe (safety plan). |
| Module 1: Increasing Biological Flexibility | The aim is to increase flexibility within biological domain biological (e.g., breathing, nutrition, sleep)  Activities include  increasing physical activity, brain and gut nutrition, breathing control, brain training, mindfulness, progressive muscle relaxation, and visual imagery. Participants are also presented with resources on sleep hygiene, alcohol and drug use, and goal setting. |
| Module 2: Increasing Emotional Flexibility | The aim is to increase emotional flexibility (e.g., increasing emotional awareness).  Activities include  adopting an objective stance, focusing awareness on the present moment, allowing emotions to take their natural course and riding the emotional wave and suppression, cognitive appraisal and acceptance. Participants are also introduced to loving kindness meditation, with an additional resource on distress tolerance. |
| Module 3: Increasing Thinking Flexibility | The aim is to increase flexibility in thinking (e.g., identifying and challenging unhelpful cognitions).  Activities include: cognitive restructuring, with additional information presented on worry, rumination and problem-solving (using the SMART technique). |
| Module 4: Increasing Behavioural Flexibility | The aim is to increase behavioural flexibility for anxiety and depression.  Activities include  reducing avoidance through gradual exposure (for anxiety) and increasing activity through behavioural activation (for depression). |
| Module 5: Increasing Wellness Flexibility | The aim is to increase wellness flexibility.  Activities include loving kindness, gratitude, personal strengths building, openness to experience, compassionate meditation, and cognitive bias modification. |
| Module 6: Increasing Life Flex-Ability | The aim is to increase overall life flexibility.  Activities include consolidation of life skills learnt and goal setting. |
| Life Flex Recharge (Booster) | The last module is a booster session which focuses on consolidation and reinforcement of learning in the Life Flex program.  Activities include relapse prevention and a 30-day challenge (choose a new increasing biological or wellness flexibility strategy, start a new hobby or revisit thought challenging, exposure or behavioural activation). |
