# Supplementary material for: Efficacy of a Digital Mental Health Biopsychosocial Transdiagnostic Intervention With or Without Therapist Assistance for Adults With Anxiety and Depression: Adaptive Randomized Controlled Trial
Source: J Med Internet Res. 2023 Jun 12;25:e45135. doi: 10.2196/45135 (PMC10337336; doi:10.2196/45135)
Supplement: Multimedia Appendix 3 [file jmir_v25i1e45135_app3.docx]

## Appendix 3

Table S2. Participant treatment preferences and allocations

|  | First Treatment Preference | Second Treatment Preference | Third Treatment Preference |
| --- | --- | --- | --- |
| ^a^ DMH (N = 42) | 10/42 (23%) | 9/42 (21%) | 23/42 (54%) |
| ^b^DMH + HI (N = 32) | 22/32 (68%) | 4/32 (12%) | 6/32 (18%) |
| ^c^DMH + LI (N = 29) | 6/ 29 (21%) | 21/29 (72%) | 2/29 (6%) |
| Total sample (N = 103) | 38/103 (36%) | 34/103 (33%) | 31/103 (30%) |

### ^a^DMH = DMH intervention program only

### ^b^DMH + HI = high-intensity therapist-assistance

### ^c^DMH + LI = low-intensity therapist-assistance
