# Supplementary material for: Efficacy of a Digital Mental Health Biopsychosocial Transdiagnostic Intervention With or Without Therapist Assistance for Adults With Anxiety and Depression: Adaptive Randomized Controlled Trial
Source: J Med Internet Res. 2023 Jun 12;25:e45135. doi: 10.2196/45135 (PMC10337336; doi:10.2196/45135)
Supplement: Multimedia Appendix 4 [file jmir_v25i1e45135_app4.docx]

## Appendix 4

Table S3. Descriptive of outcomes at baseline by conditions

| Variables | Overall sample^a^ | DMH^ac^  (N = 42) | DMH + LI  ^a d^  (N = 29) | DMH+ HI  ^a e^  (N = 32) | P-value^b^ |
| --- | --- | --- | --- | --- | --- |
| GAD-7 | 11.43 (5.16) | 10.98 (5.32) | 12.03 (4.95) | 11.47 (5.25) | .70 |
| PHQ-9 | 12.91 (5.19) | 13.14 (4.64) | 12.45 (5.72) | 13.03 (5.51) | .85 |
| Quality of life | 3.51 (1.06) | 3.43 (0.99) | 3.62 (1.21) | 3.53 (1.02) | .75 |
| Utility index | 0.55 (0.21) | 0.57 (0.18) | 0.51 (0.21) | 0.56 (0.23) | .52 |
| Social support | 2.92 (0.97) | 2.93 (0.87) | 2.86 (0.99) | 2.97 (1.09) | .91 |
| Sleeping minutes | 422.14 (76.54) | 405.83 (70.81) | 442.24 (78.42) | 425.31 (79.64) | .14 |
| Physical health | 3.35 (1.14) | 3.50 (0.99) | 3.38 (1.27) | 3.13 (1.18) | .37 |
| Mental health | 2.55 (0.74) | 2.62 (0.66) | 2.45 (0.74) | 2.56 (0.84) | .63 |

^a^ Estimated in Mean (SD)

^b^ *P*-values based on ANOVA

^c^DMH: digital mental health intervention program only

^d^ DMH + LI: low-intensity therapist-assistance

^e^ DMH + HI: high-intensity therapist-assistance
