# Supplementary material for: Efficacy of a Digital Mental Health Biopsychosocial Transdiagnostic Intervention With or Without Therapist Assistance for Adults With Anxiety and Depression: Adaptive Randomized Controlled Trial
Source: J Med Internet Res. 2023 Jun 12;25:e45135. doi: 10.2196/45135 (PMC10337336; doi:10.2196/45135)
Supplement: Multimedia Appendix 5 [file jmir_v25i1e45135_app5.docx]

## Appendix 5

Table S4. Change in intervention outcomes over time

| Variables  (N = 103) | Pre:  Week 0  Mean (SD) | Post:  Week 9  Mean (SD) | Follow-up:  Week 21  Mean (SD) | η^2c^ | Cohen’s d  Week 0 vs 9 | Cohen’s d  Week 0 vs 21 |
| --- | --- | --- | --- | --- | --- | --- |
| GAD-7 | 11.43 (5.16) | 4.86 (4.99) | 4.22 (5.38) | .51*** | 1.29***^b^ | 1.37***^b^ |
| PHQ-9 | 12.91 (5.19) | 5.15 (5.28) | 4.95 (6.08) | .57*** | 1.49***^b^ | 1.42***^b^ |
| Quality of life health rating | 3.51 (1.06) | 4.07 (0.93) | 4.19 (1.08) | .22*** | -0.56***^a^ | -0.64***^a^ |
| Quality of life utility index | 0.55 (0.21) | 0.65 (0.20) | 0.65 (0.21) | .17*** | -0.49***^b^ | -0.51***^b^ |
| Social support | 2.92 (0.97) | 3.19 (1.12) | 3.09 (1.11) | .02 | -0.27*^a^ | -0.17^a^ |
| Sleeping time (minutes) | 422.14 (76.54) | 435.75 (67.44) | 444.40 (70.40) | .05* | -0.19**^b^ | -0.30*^b^ |
| Physical health rating | 3.35 (1.14) | 3.55 (1.06) | 3.81 (1.02) | .08** | -0.18^b^ | -0.42**^b^ |
| Mental health rating | 2.55 (0.74) | 3.52 (0.93) | 3.55 (1.08) | .39*** | -1.19***^b^ | -1.15***^b^ |

^*^ *P*< 0.05, ^**^ *p* < 0.01, ^***^ *p* < 0.001

^a^ *P*-value was based on paired *t*-test

^b^ *P*-value was based on Wilcoxon signed-rank test

^c^ Effect size was based on Repeated Measures ANOVA.
