# Supplementary material for: Efficacy of a Digital Mental Health Biopsychosocial Transdiagnostic Intervention With or Without Therapist Assistance for Adults With Anxiety and Depression: Adaptive Randomized Controlled Trial
Source: J Med Internet Res. 2023 Jun 12;25:e45135. doi: 10.2196/45135 (PMC10337336; doi:10.2196/45135)
Supplement: Multimedia Appendix 6 [file jmir_v25i1e45135_app6.docx]

## Appendix 6

Figure S1. Reliable and clinically significant change in GAD-7 score among dMH intervention only program participants

**
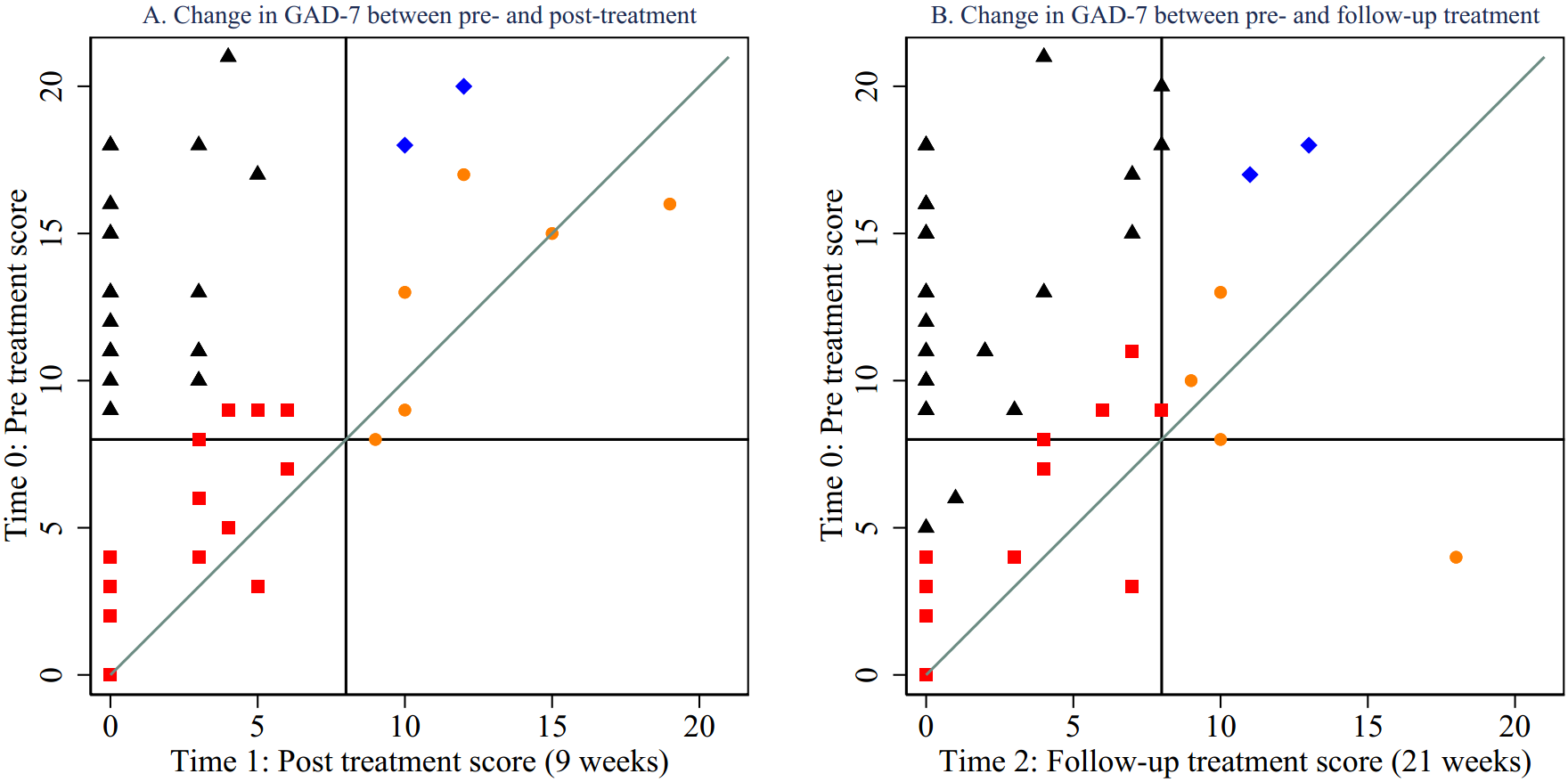
**
