# Supplementary material for: Efficacy of a Digital Mental Health Biopsychosocial Transdiagnostic Intervention With or Without Therapist Assistance for Adults With Anxiety and Depression: Adaptive Randomized Controlled Trial
Source: J Med Internet Res. 2023 Jun 12;25:e45135. doi: 10.2196/45135 (PMC10337336; doi:10.2196/45135)
Supplement: Multimedia Appendix 8 [file jmir_v25i1e45135_app8.docx]

## Appendix 8

Figure S3. Reliable and clinically significant change in GAD-7 score among high-intensity therapist-assistance participants

**
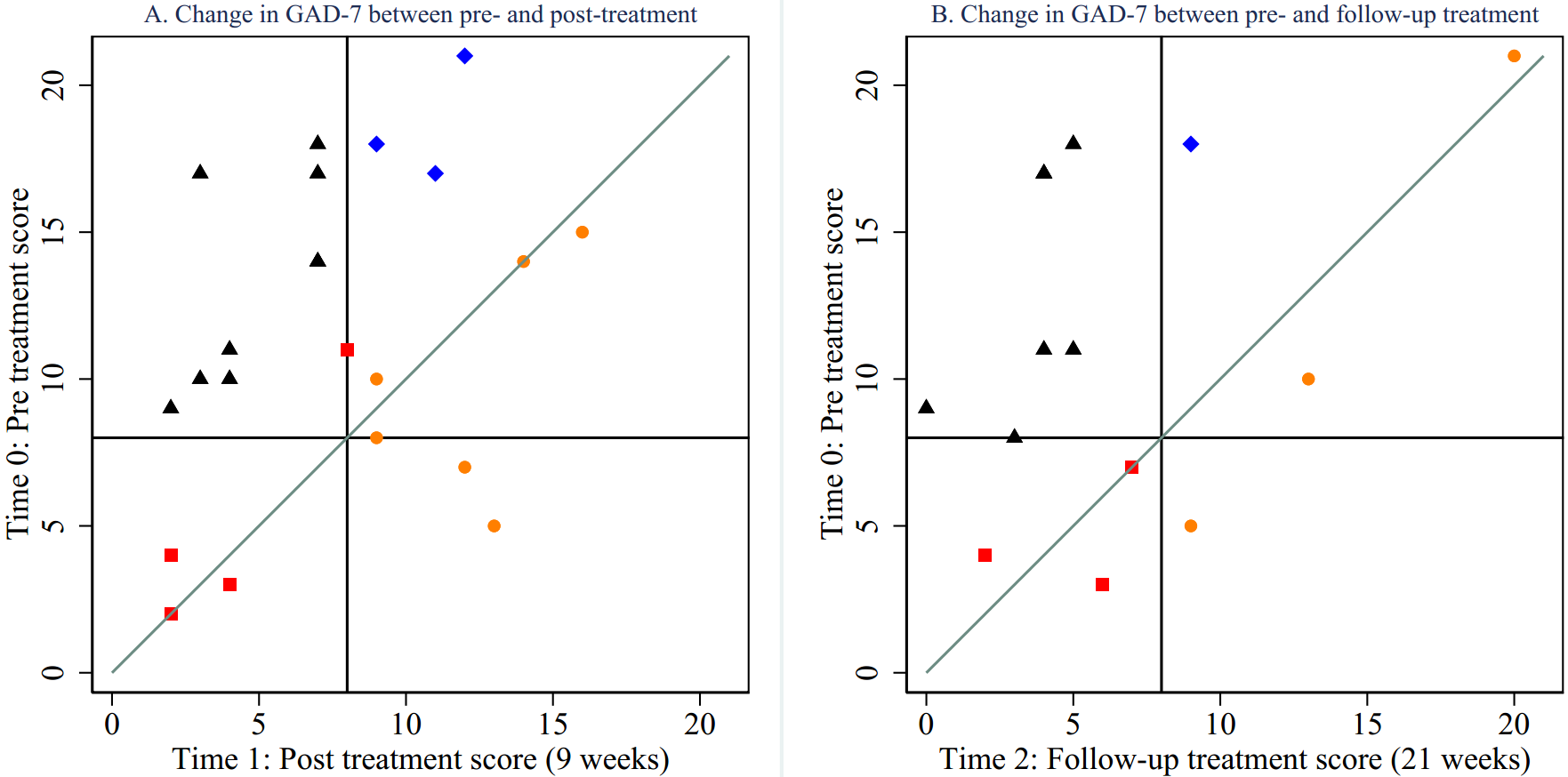
**
