# Supplementary material for: Efficacy of a Digital Mental Health Biopsychosocial Transdiagnostic Intervention With or Without Therapist Assistance for Adults With Anxiety and Depression: Adaptive Randomized Controlled Trial
Source: J Med Internet Res. 2023 Jun 12;25:e45135. doi: 10.2196/45135 (PMC10337336; doi:10.2196/45135)
Supplement: Multimedia Appendix 9 [file jmir_v25i1e45135_app9.docx]

## Appendix 9

Figure S4. Reliable and clinically significant change in PHQ-9 score among dMH intervention only program participants

**
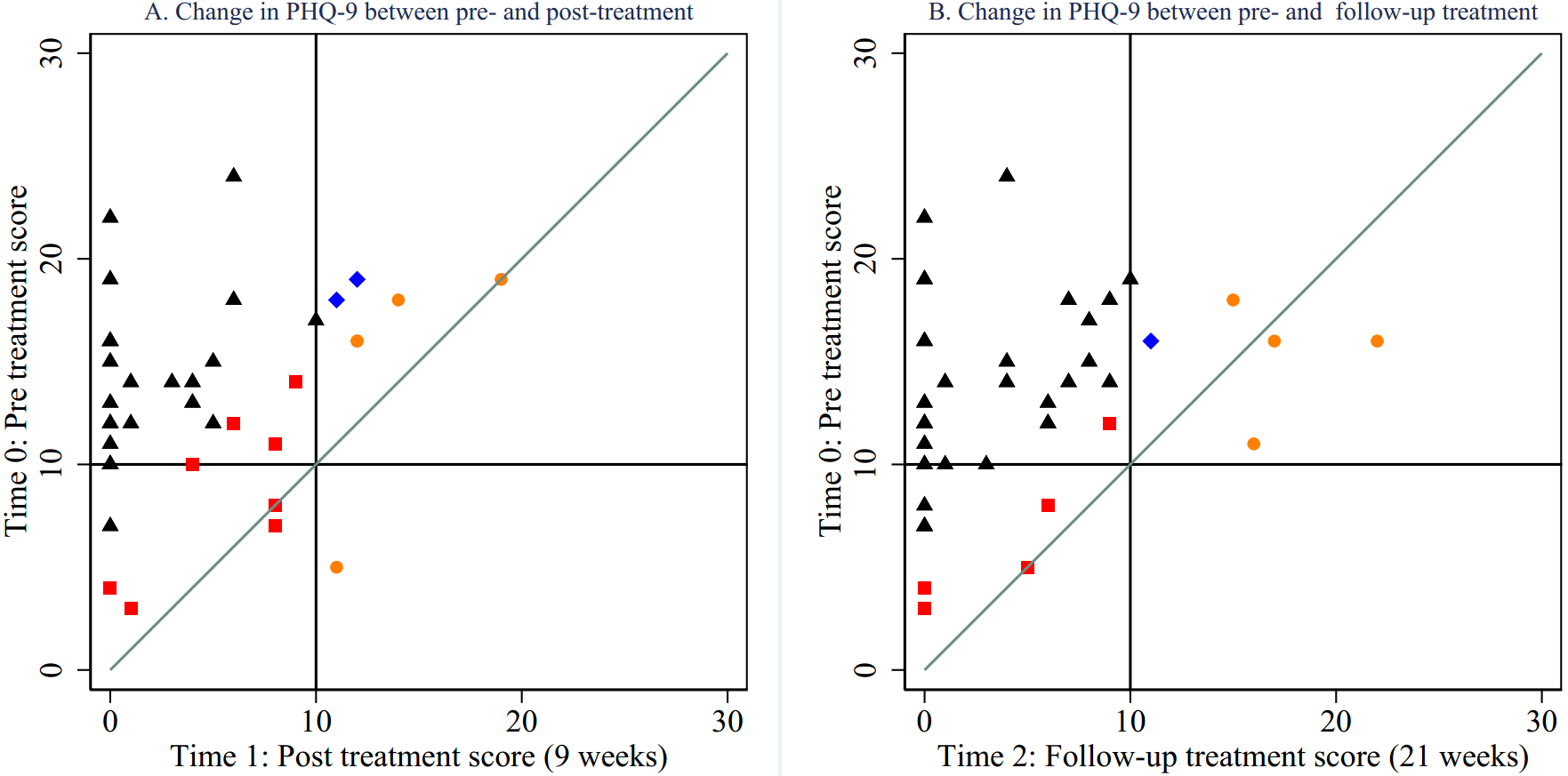
**
