# Supplementary material for: Efficacy of a Digital Mental Health Biopsychosocial Transdiagnostic Intervention With or Without Therapist Assistance for Adults With Anxiety and Depression: Adaptive Randomized Controlled Trial
Source: J Med Internet Res. 2023 Jun 12;25:e45135. doi: 10.2196/45135 (PMC10337336; doi:10.2196/45135)
Supplement: Multimedia Appendix 11 [file jmir_v25i1e45135_app11.docx]

## Appendix 11

Figure S6. Reliable and clinically significant change in PHQ-9 score among high-intensity therapist-assistance participants

**
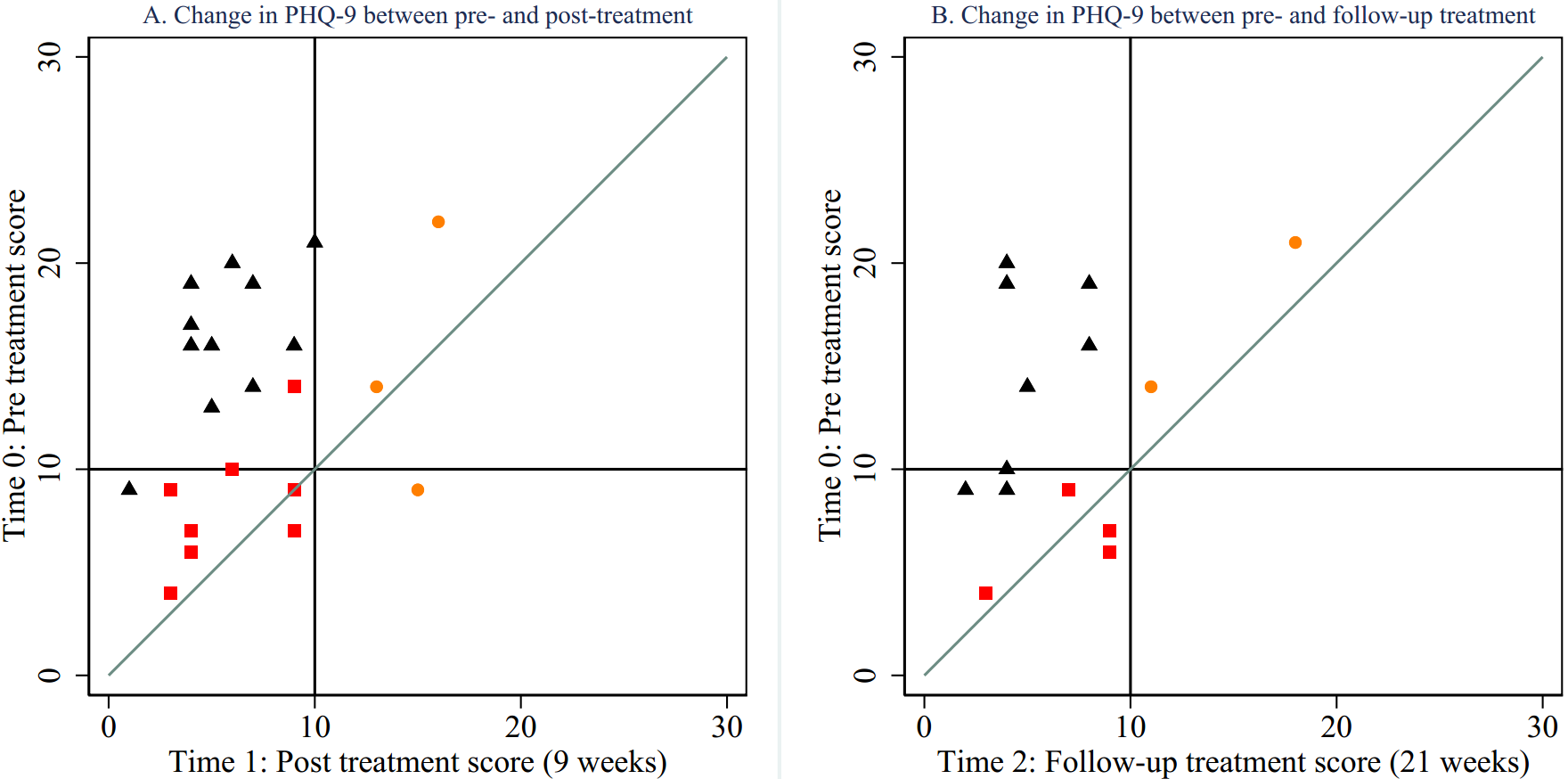
**

**Figure description:**  Change in GAD-7 (Figure S3, S4, and S5) and PHQ-9 (Figure S6, S7 and S8) between pre- and post-treatment (T0 to T1, Panel A) and pre-treatment and 21 week follow up (T0 to T2, Panel B), stratified by treatment conditions, dMH intervention program, low-intensive therapist-assistance, and high-intensive therapist-assistance. The diagonal line indicates no change. The horizontal and vertical lines indicate the GAD-7 and PHQ-9 clinical cutoff of ≥8 and ≥10, respectively pre- and post-treatment.

Orange circle: No reliable or clinically significant change

Blue diamond: Reliable, but not clinically significant change

Grey triangle: Reliable and clinically significant change

Red square: Not reliable, but clinically significant change
