# Supplementary material for: Efficacy of a Digital Mental Health Biopsychosocial Transdiagnostic Intervention With or Without Therapist Assistance for Adults With Anxiety and Depression: Adaptive Randomized Controlled Trial
Source: J Med Internet Res. 2023 Jun 12;25:e45135. doi: 10.2196/45135 (PMC10337336; doi:10.2196/45135)
Supplement: Multimedia Appendix 12 [file jmir_v25i1e45135_app12.docx]

## Appendix 12

Table S5. Correlations among outcomes and repeated measures

| Variables | GAD-7 (week 0) | GAD-7 (week 3) | GAD-7 (week 6) | GAD-7 (week 9) | GAD-7 (week 21) | PHQ-9 (week 0) | PHQ-9 (week 3) | PHQ-9 (week 6) | PHQ-9 (week 9) | PHQ-9 (week 21) |
| --- | --- | --- | --- | --- | --- | --- | --- | --- | --- | --- |
| GAD-7 (week 0) | 1 |  |  |  |  |  |  |  |  |  |
| GAD-7 (week 3) | 0.573 | 1 |  |  |  |  |  |  |  |  |
|  | 0.000 |  |  |  |  |  |  |  |  |  |
| GAD-7 (week 6) | 0.275 | 0.407 | 1 |  |  |  |  |  |  |  |
|  | 0.008 | 0.000 |  |  |  |  |  |  |  |  |
| GAD-7 (week 9) | 0.349 | 0.435 | 0.555 | 1 |  |  |  |  |  |  |
|  | 0.001 | 0.000 | 0.000 |  |  |  |  |  |  |  |
| GAD-7 (week 21) | 0.299 | 0.431 | 0.564 | 0.651 | 1 |  |  |  |  |  |
|  | 0.006 | 0.000 | 0.000 | 0.000 |  |  |  |  |  |  |
| PHQ-9 (week 0) | 0.572 | 0.272 | 0.283 | 0.265 | 0.357 | 1 |  |  |  |  |
|  | 0.000 | 0.006 | 0.006 | 0.010 | 0.001 |  |  |  |  |  |
| PHQ-9 (week 3) | 0.412 | 0.784 | 0.443 | 0.407 | 0.476 | 0.480 | 1 |  |  |  |
|  | 0.000 | 0.000 | 0.000 | 0.000 | 0.000 | 0.000 |  |  |  |  |
| PHQ-9 (week 6) | 0.190 | 0.373 | 0.857 | 0.445 | 0.544 | 0.362 | 0.556 | 1 |  |  |
|  | 0.069 | 0.000 | 0.000 | 0.000 | 0.000 | 0.000 | 0.000 |  |  |  |
| PHQ-9 (week 9) | 0.257 | 0.374 | 0.497 | 0.833 | 0.675 | 0.350 | 0.484 | 0.500 | 1 |  |
|  | 0.013 | 0.000 | 0.000 | 0.000 | 0.000 | 0.001 | 0.000 | 0.000 |  |  |
| PHQ-9 (week 21) | 0.209 | 0.450 | 0.522 | 0.571 | 0.905 | 0.395 | 0.526 | 0.641 | 0.679 | 1 |
|  | 0.055 | 0.000 | 0.000 | 0.000 | 0.000 | 0.000 | 0.000 | 0.000 | 0.000 |  |

The first line and second line in each row represents Pearson’s correlation coefficients and P-values, respectively.
