# Supplementary material for: Efficacy of a Digital Mental Health Biopsychosocial Transdiagnostic Intervention With or Without Therapist Assistance for Adults With Anxiety and Depression: Adaptive Randomized Controlled Trial
Source: J Med Internet Res. 2023 Jun 12;25:e45135. doi: 10.2196/45135 (PMC10337336; doi:10.2196/45135)
Supplement: Multimedia Appendix 13 [file jmir_v25i1e45135_app13.docx]

## Appendix 13

Table S6. Remainder of the socio-demographic and clinical characteristics of the sample at baseline by intervention conditions

| Variables | Overall^a^  (N =103) | DMH^a,c^  (N = 42) | DMH + LI^a, d^  (N = 29) | DMH + HI^a, e^  (N = 32) | *P*-value |
| --- | --- | --- | --- | --- | --- |
| **No of mild exercise minutes**, mean (SD) | 195.23 (212.5) | 185.86 (169.7) | 204.93 (231.3) | 198.75 (249.1) | .93^b^ |
| **No of moderate exercise minutes**, mean (SD) | 70.0 (129.9) | 57.79 (76.2) | 80.0 (140.4) | 77.01 (172.3) | .73^b^ |
| **No of vigorous exercise minutes**, mean (SD) | 68.8 (152.9) | 36.48 (71.1) | 78.9 (187.6) | 102.0 (189.2) | .17^b^ |
| **Drink alcohol** |  |  |  |  | .83 |
| Never | 13 (12%) | 6 (14%) | 2 (6%) | 5 (15%) |  |
| Monthly or less | 31 (30%) | 12 (28%) | 9 (31%) | 10 (31%) |  |
| 2-4 times a month | 32 (31%) | 13 (31%) | 8 (27%) | 11 (34%) |  |
| ≥2 times a week | 27 (26%) | 11 (26%) | 10 (34%) | 6 (18%) |  |
| **Illicit drug use** |  |  |  |  | .21 |
| Never | 41 (39%) | 21 (50%) | 7 (24%) | 13 (40%) |  |
| Not in the last 12 months | 33 (32%) | 10 (23%) | 15 (51%) | 8 (25%) |  |
| No more than 12 times in the 12 months | 21 (20%) | 8 (19%) | 5 (17%) | 8 (25%) |  |
| ≥2 times a month | 8 (7%) | 3 (7%) | 2 (6%) | 3 (9%) |  |
| **Insomnia** |  |  |  |  | .86 |
| None | 18 (17%) | 9 (21%) | 4 (13%) | 5 (15%) |  |
| Mild | 25 (24%) | 8 (19%) | 9 (31%) | 8 (25%) |  |
| Moderate | 31 (30%) | 12 (28%) | 8 (27%) | 11 (34%) |  |
| Severe | 20 (19%) | 8 (19%) | 7 (24%) | 5 (15%) |  |
| Very severe | 9 (8%) | 5 (11%) | 1 (3%) | 3 (9%) |  |
| **Difficulty sleeping** |  |  |  |  | .96 |
| None | 18 (17%) | 8 (19%) | 3 (10%) | 7 (21%) |  |
| Mild | 33 (32%) | 15 (35%) | 9 (31%) | 9 (28%) |  |
| Moderate | 34 (33%) | 12 (28%) | 12 (41%) | 10 (31%) |  |
| Severe | 15 (14%) | 6 (14%) | 4 (13%) | 5 (15%) |  |
| Very severe | 3 (2%) | 1 (2%) | 1 (3%) | 1 (3%) |  |
| **Severity of waking up to early** |  |  |  |  | .44 |
| None | 30 (29%) | 12 (28%) | 6 (20%) | 12 (37%) |  |
| Mild | 31 (30%) | 13 (31%) | 8 (27%) | 10 (31%) |  |
| Moderate | 22 (21%) | 11 (26%) | 9 (31%) | 2 (6%) |  |
| Severe | 17 (16%) | 5 (11%) | 5 (17%) | 7 (21%) |  |
| Very severe | 3 (2%) | 1 (2%) | 1 (3%) | 1 (3%) |  |
| **Satisfaction with current sleep pattern** |  |  |  |  | .28 |
| Very dissatisfied | 18 (17%) | 9 (21%) | 4 (13%) | 5 (15%) |  |
| Dissatisfied | 38 (36%) | 11 (26%) | 14 (48%) | 13 (40%) |  |
| Moderately satisfied | 28 (27%) | 15 (35%) | 8 (27%) | 5 (15%) |  |
| Satisfied | 16 (15%) | 5 (11%) | 3 (10%) | 8 (25%) |  |
| Very satisfied | 3 (2%) | 2 (4%) | 0 (0%) | 1 (3%) |  |
| **Medication prescription** |  |  |  |  | .54 |
| No | 55 (53%) | 21 (50%) | 18 (62%) | 16 (50%) |  |
| Yes | 48 (46%) | 21 (50%) | 11 (37%) | 16 (50%) |  |
| **Access to health services** |  |  |  |  | .91 |
| Never | 3 (2%) | 2 (4%) | 1 (3%) | 0 (0%) |  |
| Accessed before but not the last 12 months | 13 (12%) | 6 (14%) | 3 (10%) | 4 (12%) |  |
| Not last 4 weeks, but the last 12 months | 47 (45%) | 17 (40%) | 14 (48%) | 16 (50%) |  |
| Yes, currently | 40 (38%) | 17 (40%) | 11 (37%) | 12 (37%) |  |
| **Access to mental health services** |  |  |  |  | .14 |
| Never | 19 (18%) | 8 (19%) | 4 (13%) | 7 (21%) |  |
| Yes, but not the last 12 months | 54 (52%) | 19 (45%) | 13 (44%) | 22 (68%) |  |
| Not currently, but the last 12 months | 23 (22%) | 11 (26%) | 9 (31%) | 3 (9%) |  |
| Yes, last 4 weeks only (pre-trial) | 7 (6%) | 4 (9%) | 3 (10%) | 0 (0%) |  |

^a^ Estimated in n (%) or Mean (SD)

^b^ *P*-values based on ANOVA; the remaining based on χ^2^.

^c^DMH: DMH intervention program only

^d^DMH + LI: low intensity therapist-assistance

^e^DMH + HI: high intensity therapist-assistance
