# Supplementary material for: Efficacy of a Digital Mental Health Biopsychosocial Transdiagnostic Intervention With or Without Therapist Assistance for Adults With Anxiety and Depression: Adaptive Randomized Controlled Trial
Source: J Med Internet Res. 2023 Jun 12;25:e45135. doi: 10.2196/45135 (PMC10337336; doi:10.2196/45135)
Supplement: Multimedia Appendix 14 [file jmir_v25i1e45135_app14.docx]

## Appendix 14

Table S7. Reliable and clinically significant change in intervention primary outcomes over times across intervention conditions

| **Variables** | **Reliable and clinically significant change**  **Week 0 vs 9**  **n (%)** | **Reliable and clinically significant change**  **Week 0 vs 21**  **n (%)** |
| --- | --- | --- |
| **GAD-7** | **31/74 (41.9)** | **32/62 (51.6)** |
| DMH^a^ | 14/35 (40.0) | 18/35 (51.4) |
| DMH + LI^b^ | 17/28 (60.7) | 17/24 (70.8) |
| DMH + HI^c^ | 8/21 (38.1) | 6/13 (46.2) |
| **PHQ-9** | **34/69 (49.3)** | **39/64 (60.9)** |
| DMH^a^ | 19/33 (57.6) | 23/33 (69.7) |
| DMH + LI^b^ | 13/27 (84.1) | 16/25 (64.0) |
| DMH + HI^c^ | 11/22 (50.0) | 8/14 (57.1) |

### ^a^DMH = DMH intervention program only

### ^b^DMH + HI = high-intensity therapist-assistance

### ^c^DMH + LI = low-intensity therapist-assistance
