# Supplementary material for: Efficacy of a Digital Mental Health Biopsychosocial Transdiagnostic Intervention With or Without Therapist Assistance for Adults With Anxiety and Depression: Adaptive Randomized Controlled Trial
Source: J Med Internet Res. 2023 Jun 12;25:e45135. doi: 10.2196/45135 (PMC10337336; doi:10.2196/45135)
Supplement: Multimedia Appendix 16 [file jmir_v25i1e45135_app16.docx]

## Appendix 16

Figure S8. Distribution of the individual PHQ-9 change by reliable change status in different timepoints

**
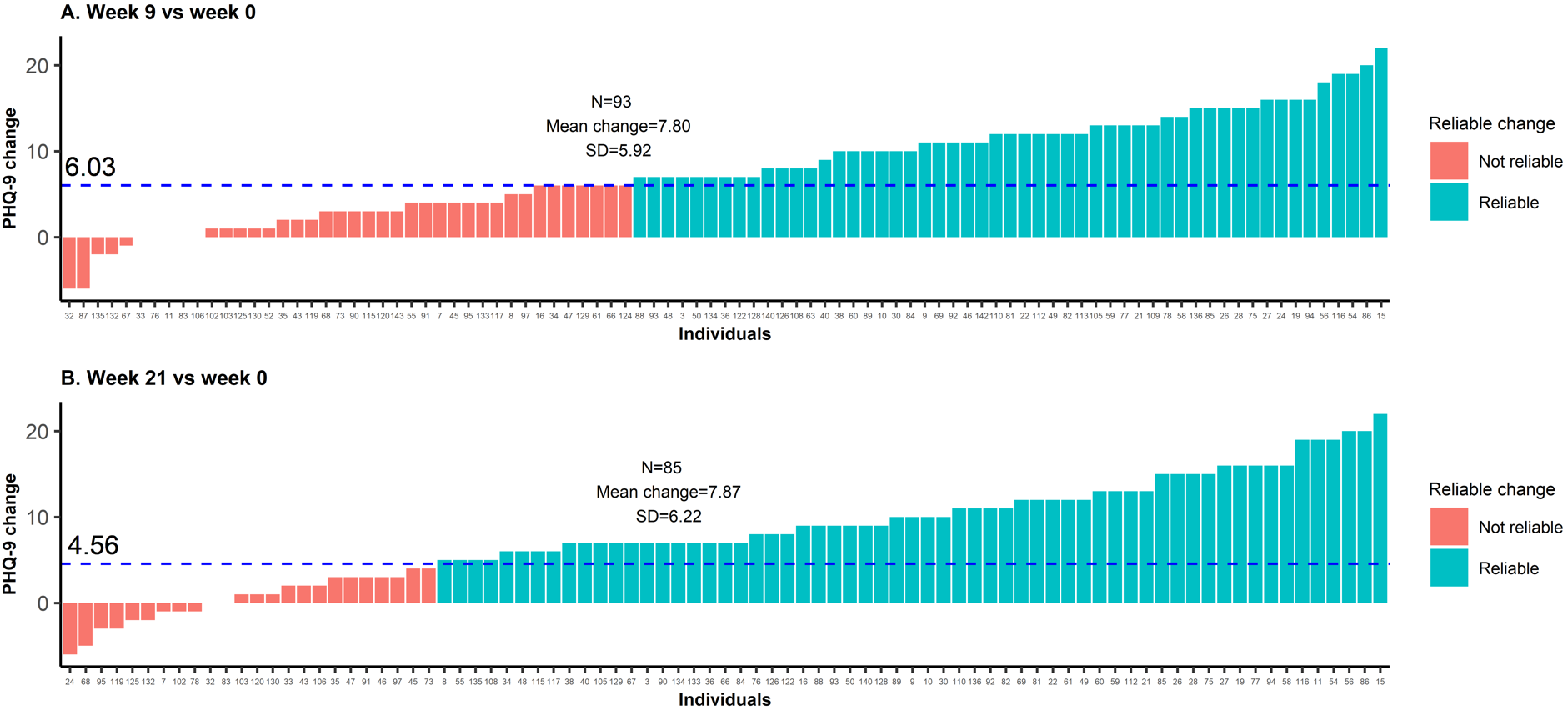
**
