# Supplementary material for: Efficacy of a Digital Mental Health Biopsychosocial Transdiagnostic Intervention With or Without Therapist Assistance for Adults With Anxiety and Depression: Adaptive Randomized Controlled Trial
Source: J Med Internet Res. 2023 Jun 12;25:e45135. doi: 10.2196/45135 (PMC10337336; doi:10.2196/45135)
Supplement: Multimedia Appendix 17 [file jmir_v25i1e45135_app17.docx]

## Appendix 17

Table S8. Frequency of each disorder at pre-intervention, post-intervention and follow-up

|  | Pre-Intervention  _____________________ | | | Post-Intervention  ______________________ | | | 3-Month Follow-up  _______________________ | | |
| --- | --- | --- | --- | --- | --- | --- | --- | --- | --- |
|  | DMH ^f^  (N = 42,%) | DMH + LI ^g^  (N = 29,%) | DMH + HI ^h^ (N = 32,%) | DMH ^f^ (N = 27,%) | DMH + LI ^g^ (N = 18,%) | DMH + HI ^h^ (N = 20,%) | DMH ^f^  (N = 22,%) | DMH + LI ^g^ (N = 13,%) | DMH + HI ^h^  (N = 14,%) |
| **MDD**^a^ | 13 (30) | 8 (27) | 6 (18) | 0 (0) | 1 (5) | 0 (0) | 1 (4) | 2 (15) | 0 (0) |
| **Sub-threshold MDD**^a^ | 0 (0) | 1 (3) | 0 (0) | 2 (7) | 2 (11) | 0 (0) | 0 (0) | 0 (0) | 2 (14) |
| **MDD**^a^ **comorbid with anx** ^b^ | 23 (54) | 14 (48) | 20 (62) | 2 (7) | 1 (5) | 1 (5) | 1 (4) | 0 (0) | 0 (0) |
| **Sub-threshold MDD**^a^ **comorbid with anx** ^b^ | 0 (0) | 0 (0) | 0 (0) | 1 (3) | 0 (0) | 0 (0) | 0 (0) | 0 (0) | 1 (7) |
| **GAD** ^c^ | 1 (2) | 1 (3) | 0 (0) | 1 (3) | 1 (5) | 0 (0) | 0 (0) | 0 (0) | 0 (0) |
| **SAD** ^d^ | 1 (2) | 1 (3) | 1 (3) | 0 (0) | 1 (5) | 4 (2) | 0 (0) | 1 (7) | 0 (0) |
| **Panic** ^e^ | 0 (0) | 0 (0) | 0 (0) | 0 (0) | 0 (0) | 0 (0) | 1 (4) | 0 (0) | 1 (7) |
| **Agoraphobia** | 0 (0) | 0 (0) | 0 (0) | 0 (0) | 0 (0) | 0 (0) | 0 (0) | 0 (0) | 0 (0) |
| **Comorbid anx** ^b^ | 4 (9) | 4 (13) | 5 (15) | 0 (0) | 2 (11) | 1 (5) | 0 (0) | 1 (7) | 0 (0) |
| **Sub-threshold anx** ^b^ | 0 (0) | 0 (0) | 0 (0) | 0 (0) | 0 (0) | 0 (0) | 0 (0) | 0 (0) | 0 (0) |
| **Other** | 0 (0) | 0 (0) | 0 (0) | 1 (3) | 0 (0) | 0 (0) | 1 (4) | 0 (0) | 0 (0) |
| **No criteria met** | 0 (0) | 0 (0) | 0 (0) | 20 (74) | 10 (55) | 14 (70) | 15 (68) | 9 (69) | 10 (71) |
| **Did not attend (of total n)** | 0 (0) | 0 (0) | 0 (0) | 15 (35) | 11 (37) | 12 (37) | 20 (47) | 20 (47) | 20 (47) |

^a^MDD: Major Depressive Disorder

^b^Anx: anxiety

^c^GAD: Generalised Anxiety Disorder

^d^SAD: Social Anxiety Disorder

^e^Panic: Panic Disorder

^f^DMH: DMH intervention program only

^g^DMH + LI: low-intensity therapist-assistance

^h^DMH + HI: high-intensity therapist-assistance
