# Supplementary material for: Efficacy of a Digital Mental Health Biopsychosocial Transdiagnostic Intervention With or Without Therapist Assistance for Adults With Anxiety and Depression: Adaptive Randomized Controlled Trial
Source: J Med Internet Res. 2023 Jun 12;25:e45135. doi: 10.2196/45135 (PMC10337336; doi:10.2196/45135)
Supplement: Multimedia Appendix 18 [file jmir_v25i1e45135_app18.docx]

## Appendix 18

Table S9. Treatment satisfaction ratings

| Item | N | % |
| --- | --- | --- |
| 1. How would you rate the quality of treatment delivered by Life Flex?   Good to excellent | 57/67 | 85% |
| 1. Did you get the kind of information/strategies you wanted?   Yes, definitely | 61/67 | 91% |
| 1. To what extent has Life Flex met your needs?   Almost all of my needs have been met | 60/67 | 89.5% |
| 1. If a friend were in need of a similar assistance, would you recommend Life Flex to him or her?   Yes, definitely | 59/67 | 88.1% |
| 1. How satisfied are you with the amount of information you have received?   Very satisfied | 62/67 | 92.5% |
| 1. Has Life Flex helped you to deal more effectively with your problems?   Yes, it helped a great deal | 55/67 | 82.1% |
| 1. In an overall, general sense, how satisfied are you with Life Flex?   Very satisfied | 56/67 | 83.6% |
| 1. If you were to seek help, would you come back to Life Flex?   Yes, definitely | 55/67 | 82.1% |
